# An improved assembly of the “Cascade” hop (*Humulus lupulus*) genome uncovers signatures of molecular evolution and refines time of divergence estimates for the Cannabaceae family

**DOI:** 10.1093/hr/uhac281

**Published:** 2022-12-07

**Authors:** Lillian K Padgitt-Cobb, Nicholi J Pitra, Paul D Matthews, John A Henning, David A Hendrix

**Affiliations:** Department of Biochemistry and Biophysics, Oregon State University, Corvallis, Oregon, USA; Department of Research and Development, Hopsteiner, S.S. Steiner, Inc., 1 West Washington Avenue, Yakima, Washington 98903, USA; Department of Research and Development, Hopsteiner, S.S. Steiner, Inc., 1 West Washington Avenue, Yakima, Washington 98903, USA; Forage Seed and Cereal Research Unit, USDA-ARS, Corvallis, Oregon, USA; Department of Crop and Soil Science, Oregon State University, Corvallis, Oregon, USA; Department of Biochemistry and Biophysics, Oregon State University, Corvallis, Oregon, USA; School of Electrical Engineering and Computer Science, Oregon State University, Corvallis, Oregon, USA

## Abstract

We present a chromosome-level assembly of the Cascade hop (*Humulus lupulus* L. var. *lupulus*) genome. The hop genome is large (2.8 Gb) and complex, and early attempts at assembly were fragmented. Recent advances have made assembly of the hop genome more tractable, transforming the extent of investigation that can occur. The chromosome-level assembly of Cascade was developed by scaffolding the previously reported Cascade assembly generated with PacBio long-read sequencing and polishing with Illumina short-read DNA sequencing. We developed gene models and repeat annotations and used a controlled bi-parental mapping population to identify significant sex-associated markers. We assessed molecular evolution in gene sequences, gene family expansion and contraction, and time of divergence from *Cannabis sativa* and other closely related plant species using Bayesian inference. We identified the putative sex chromosome in the female genome based on significant sex-associated markers from the bi-parental mapping population. While the estimate of repeat content (~64%) is similar to the estimate for the hemp genome, syntenic blocks in hop contain a greater percentage of LTRs. Hop is enriched for disease resistance-associated genes in syntenic gene blocks and expanded gene families. The Cascade chromosome-level assembly will inform cultivation strategies and serve to deepen our understanding of the hop genomic landscape, benefiting hop researchers and the Cannabaceae genomics community.

## Introduction

**Figure 1:**
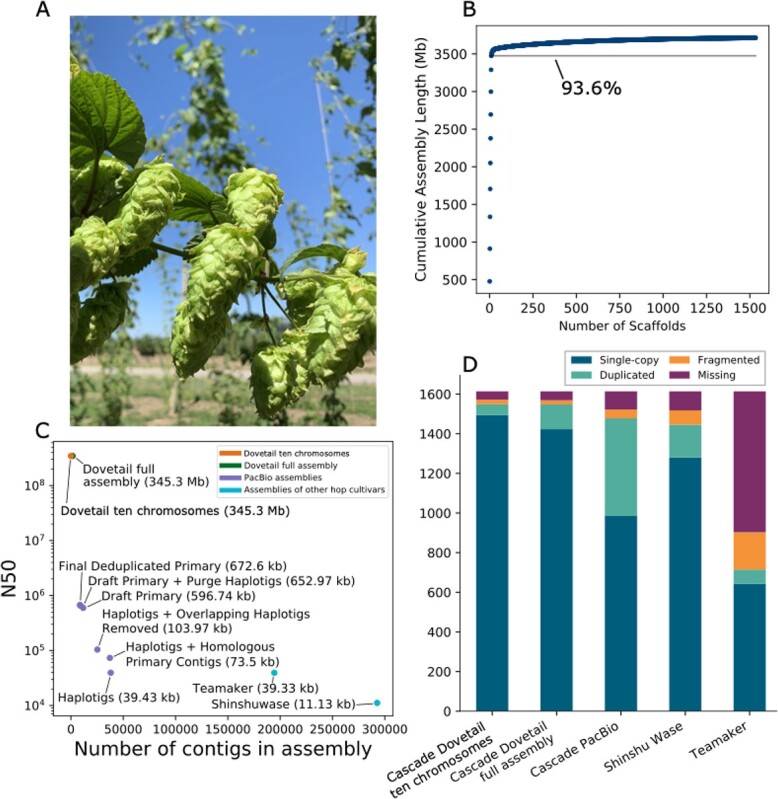
Analysis of Cascade Hop Dovetail assembly. **A)** Image of hop cones. **B)** Scatter plot depicting the cumulative assembly length on the y-axis relative to the number of scaffolds on the x-axis, showing that 93.6% of the assembly is contained in the largest 10 scaffolds. **C)** Scatter plot showing the number of contigs or scaffolds in each assembly on the x-axis versus the N50 on the y-axis. **D)** BUSCO result comparison among the assemblies for all Dovetail scaffolds; the 10 largest scaffolds in the Dovetail assembly; PacBio Cascade; Shinshu Wase; and Teamaker.

Hop (*Humulus lupulus* L. var. *lupulus*) is a diploid (2n = 18 + XX/XY), wind-pollinated, perennial plant [1, 2] with cultural, economic, and pharmacological significance, including use in brewing and consumables for flavor and aroma. *H. lupulus* is typically dioecious, having male and female plants, although monoecious individuals also occur [3]. The female inflorescences, or cones, of hop plants are known as “hops,” and contain lupulin glands (glandular trichomes), which are the primary site of synthesis and storage of resins, bitter acids, essential oils, and flavonoids [4–6] (Figure 1A).

Hop cultivar Cascade is known for its floral and citrus aroma and is the most widely produced American “aroma” hop [7]. Cascade was developed at Oregon State University and USDA [8, 9]. The pedigree of Cascade is (Fuggle x [Serebrianka x Fuggle-seedling]) x open-pollinated seed [10]. The oil content of Cascade is rich in myrcene (45–60% of oil content), cohumulone (33–40% of oil content), and humulene (8–13% of oil content) [7]. Linalool and geraniol also contribute to the flavor and aroma of Cascade [11].

Hop grows optimally between the 35° and 55° latitude in the Northern and Southern hemispheres [12, 13]; however, lower latitudes can also support hop production [13]. Hop is susceptible to fungal diseases, including powdery mildew (*Podosphaera macularis*), downy mildew (*Pseudoperonospora humuli* (Miyabe & Takah.) G.W. Wilson), and viruses [13].

*Humulus* is part of the Cannabaceae family, along with *Cannabis*. *Humulus* and *Cannabis* form a sister clade to the clade containing *Celtis*, *Trema*, and *Parasponia. Humulus* includes *H. lupulus* L., *H. yunnanensis* Hu., and *H. japonicus* Siebold & Zucc. (synonymous with *Humulus scandens* (Lour.) Merr. [14]. We will refer to *H. scandens* herein as *Humulus japonicus*.

The estimated date of divergence of *Humulus* and *Cannabis* is a source of debate [15, 16]. Unraveling the complex evolutionary history of species in the Cannabaceae has been impeded by sparse fossil evidence and genomic resources. With the development of new genomic data, previously estimated divergence dates can be re-evaluated and refined [17–20].

Phylogenetic analyses of modern and ancient *Cannabis* samples revealed that *H. japonicus* is more closely related to both ancient and modern samples of *Cannabis*, than *H. lupulus* [21, 22]. Successful grafting between *C. sativa*, *H. japonicus*, and *H. lupulus* further underscores the close relationship between these species [23]. The xanthohumol and bitter acid pathways in hop and the cannabinoid pathway in *Cannabis* contain enzymes that perform analogous reactions and accept similar precursor structures [24], which is important for interpreting the evolution of genes involved in these pathways. For all three pathways, the first reaction involves type III polyketide synthases and malonyl-CoA, and the second reaction involves aromatic prenyltransferases and isoprenoid structures.

The hop genome is large and heterozygous, and the size, complexity, and repeat content of the hop genome hindered previous assembly efforts [25, 26]. Long-read sequencing and haplotype-aware assembly algorithms improved the resolution of the hop genome [27]. However, even with long-read sequencing, the assembly remained fragmented (N50 ∼ 673 kb) and contig order was unknown. Contiguous genome sequences are necessary for investigating synteny and genomic organization.

Scaffolding with Dovetail high-throughput chromatin conformation capture (Hi-C) libraries allows detection of long-range DNA interactions by sequencing fragments of cross-linked chromatin, providing information about spatial organization of DNA [28]. Based on Hi-C long-range information, the PacBio long-read contigs were ordered and oriented into chromosome-level scaffolds. Here we describe an improved assembly of the Cascade female genome developed with the draft, phased PacBio long-read assembly of Cascade [27], along with accompanying analyses of genome content, organization, and evolution. The development of cultivars with enhanced tolerance to abiotic and biotic stresses and distinct flavor and aroma profiles is a research area of priority [13] and will be benefitted by the development of the improved genome assembly of Cascade.

## Results

### Genome sequencing and assembly

The size of the PacBio primary assembly used to anchor the scaffolds is 3 711 963 939 bp, and the resulting Dovetail Hi-C assembly is 3 712 781 139 bp (Supplementary Data Table S1). The estimated physical coverage for Dovetail is 37.02X (Supplementary Data Table S1). The library insert size distribution is shown in Supplementary Data Figure S1, and the link density histogram for read position versus mate position is shown in Supplementary Data Figure S2. The polished Dovetail assembly is 3 713 677 344 bp (Supplementary Data Table S2).

The N50 increased from 673 kb for PacBio to 345.208 Mb for Dovetail (Figure 1C; Supplementary Data Table S3). The scaffold N90 increased from 221 kb for PacBio to 185.170 Mb for Dovetail (Supplementary Data Figure S3). The 10 largest Dovetail scaffolds have a total length of 3.47 Gb, and 93.6% of the assembly is represented in the 10 largest scaffolds (Figure 1B). Our downstream analyses focus on the 10 largest scaffolds, which approximate the expected 10 chromosomes of the hop genome. In figures and tables, we designate the 10 largest scaffolds as chromosomes 1–10, which are labeled in descending order according to length, except for the putative X chromosome, which we designate as chromosome X.

GC content of the full, polished Dovetail assembly is 39.13% and 0.022% Ns. Nucleotide content in the largest 10 scaffolds is shown in Supplementary Data Figure S4A. Among dinucleotides, CG content is depleted (Supplementary Data Figure S4B). Expected versus observed frequency of CHG and CHH trinucleotides, where H represents A, C, or T nucleotides, reveals an enrichment of CHH trinucleotides (Supplementary Data Figure S4C). CHG and CHH trinucleotides are associated with DNA methylation, which is involved in gene regulation of essential plant processes, including growth and development [129].

### Assembly completeness with BUSCO

Assembly BUSCO statistics improved after polishing, from 92.0% total complete to 95.9% (Supplementary Data Table S4). In the polished, repeat-masked Dovetail assembly, restricting the BUSCO analysis to the largest 10 scaffolds reduced the percentage of duplicated BUSCOs from 7.7% to 3.4% while increasing the percentage of single-copy from 88.2% to 92.6%. Using the Viridiplantae database, the number of total complete BUSCOs is consistent with the assembly of hemp cultivar CBDRx, for which 97% of complete BUSCOs were identified [30]. Based on the inclusion of fewer duplicated BUSCO genes in the largest 10 scaffolds, we restricted our downstream synteny analyses to the largest 10 scaffolds. Figure 1D shows a comparison of the chromosome-level assembly with the PacBio Cascade primary assembly [27], Shinshu Wase assembly [26], and Teamaker assembly [35].

### Genome size, repeat content, and heterozygosity

**Figure 2:**
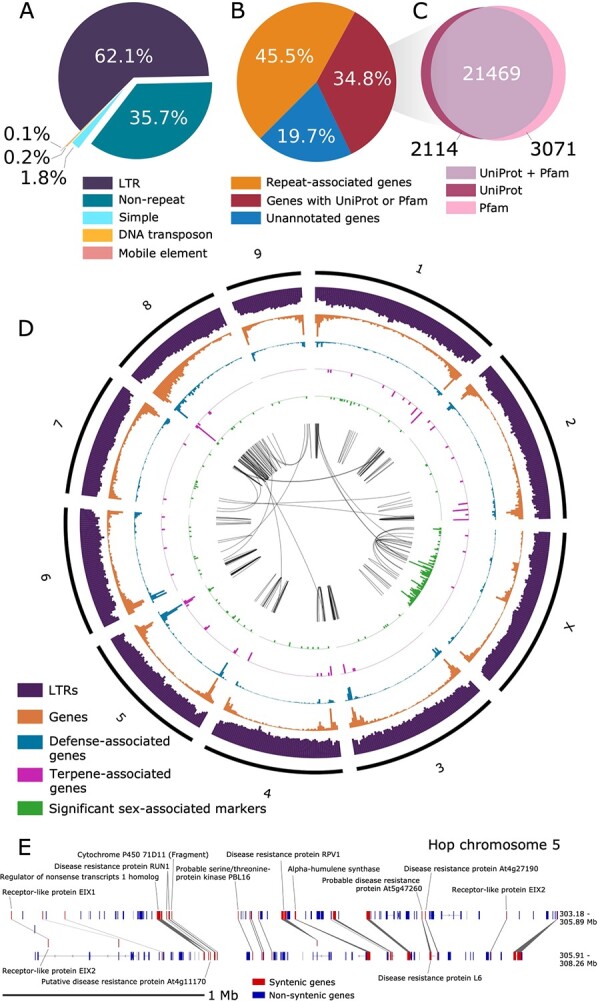
Gene and repeat content in the 10 largest assembly scaffolds. **A)** Pie chart showing percentages of different categories of repeat content relative to total repeat content. **B)** Pie chart showing the percentage of genes with similarity to a repeat-associated UniProt protein or Pfam domain, the percentage of genes with similarity to a non-repeat-associated UniProt gene or Pfam domain, and genes lacking similarity to any known UniProt protein or Pfam domain. **C)** Venn diagram showing the intersection of genes that have similarity to a UniProt gene and/or a Pfam domain. **D)** Circos plot for the largest 10 scaffolds in the Dovetail assembly showing histograms for genomic features. All histograms are split into 5 megabase (Mb) bins, depicting counts per 5 Mb, including gene density (orange; y-axis range: 4–253), putative defense response-associated genes (blue; y-axis range: 0–104), putative terpene-associated genes (pink; y-axis range: 0–8), long-terminal retrotransposons (purple; y-axis range: 51–5707), and significant sex-associated SNPs (green; y-axis range: 0–12; p-value <0.05). The center track depicts syntenic blocks within the same scaffold and across different scaffolds. **E)** Syntenic block on Dovetail chromosome 5 containing putative disease-response-associated genes and one copy of an alpha-humulene synthase. Syntenic genes are shown in red and non-syntenic genes are shown in blue. In the top track, the genomic coordinates for the first mRNA start position and last mRNA stop position are 303.18–305.89 Mb, and the analogous coordinates in the second block are 305.91–308.26 Mb.

The haploid size of the genome of *Humulus lupulus var. lupulus* estimated by flow cytometry ranges from 2.57 Gb [26] to 2.989 Gb [31] for different cultivars (Supplementary Data Table S5). Based on a k-mer distribution analysis, we estimate a haploid genome size of 3 058 114 149 bp (3.058 Gb) for Cascade. The genome is ~4.59%–5.47% heterozygous with a read error rate of ~0.48% (Supplementary Data Table S6). Out of 563 456 691 total DNA short-reads, 561 688 517 DNA short-reads (99.69%) map to the Dovetail assembly. The Dovetail assembly is 64.25% composed of repeat sequences, including 62.14% LTRs, 0.19% DNA transposons, 1.76% simple repeats, and 0.03% LINE repeats (Figure 2A, Supplementary Data Table S7).

**Figure 3:**
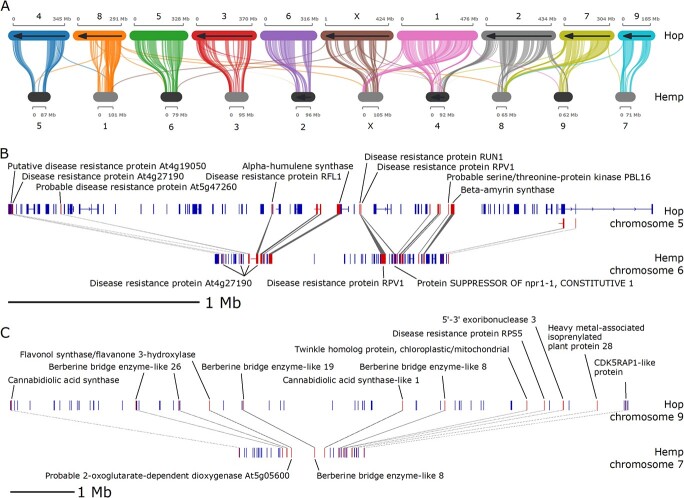
Comparative analysis of hop and hemp syntenic blocks. **A)** Syntenic blocks shared between the largest 10 scaffolds in the hop genome assembly and the largest 10 scaffolds in the hemp genome assembly. **B)** Single syntenic block between hop chromosome 5 and hemp chromosome 6 containing putative disease-response-associated genes and one copy of an alpha-humulene synthase. **C)** Single syntenic block between hop chromosome 9 and hemp chromosome 7 containing putative cannabinoid synthesis pathway genes.

### Development of linkage map

The genetic map for mapping population 2 017 014 produced 10 linkage groups, including a total of 4090 markers and an overall length of 1269.5 cM (Supplementary Data Table S8). Average genetic distance between markers is 0.35 cM with an average of 409 markers per linkage group. Chromosome 6 contains the fewest number of markers (209) while chromosome 7 has the most (627), reflecting the density of linkage disequilibrium bins formed for each chromosome, as illustrated by average gap size (Supplementary Data Table S8). Supplementary Data Figure S5 shows the association between marker positions on the genetic map versus marker location on the physical map for the 10 largest scaffolds. The genetic and linkage disequilibrium (LD) maps are provided as separate supplementary files (the genetic map is Supplementary Data Table S9 and the LD map is Supplementary Data Table S10).

### Gene model content

We generated gene models using both Transdecoder and MAKER. In cases where Transdecoder and MAKER found overlapping gene models based on exon coordinates in the same strand, we assigned priority to the Transdecoder gene model, since the Transdecoder algorithm is directly based on expression from RNA-seq (see Supplementary Data Table S11 for details about Transdecoder gene models). In cases where MAKER identified a gene model and there was no overlapping Transdecoder gene model, we included the MAKER gene model. Among the Transdecoder gene models, 94.9% are present on the largest 10 scaffolds. We assessed the completeness of gene models with BUSCO (Supplementary Data Table S12).

Identification and removal of repeat-associated gene models was based on similarity to Pfam domains and UniProt genes (Figure 2B; Supplementary Data Table S13; Supplementary Data Table S14; Supplementary Data Table S15). We identified 23 583 genes with similarity to a UniProt Embryophyta gene; 20 877 (88.53%) of these genes are present on the 10 largest Dovetail scaffolds (Figure 2C). The set of MAKER gene models has fewer gene models with high percent similarity to UniProt genes than Transdecoder (Supplementary Data Figure S6). After excluding repeat-associated gene models, as well as MAKER gene models without similarity to known genes or protein domains, 30 404 gene models were used for downstream orthology, synteny, and evolution analyses. Genes with GO terms are provided in Supplementary Data Table S16 [32]. Among the 3003 genes with putative defense or disease response-associated genes in the full assembly, 2667 of these genes occur in the largest 10 scaffolds.

We also evaluated our gene models based on orthology by comparing protein sequences of hop to seven other plant species, including *Cannabis sativa*, *Morus notabilis*, *Parasponia andersonii*, *Prunus persica*, *Trema orientale*, *Vitis vinifera*, and *Ziziphus jujuba*. A total of 22 739 orthologous gene groups (OGGs) were identified with OrthoFinder [33] (Supplementary Data Figure S11). There are 24 513 hop genes from the 10 largest scaffolds in OGGs; 58.3% of OGGs contain hop genes, and 10.5% of hop genes are in species-specific OGGs.

### Density of genes, long terminal retrotransposons (LTRs), and sex-associated markers

We visualized the density of genes and LTRs across the 10 largest scaffolds in a circos plot (Figure 2D). For most scaffolds, gene density is higher at the ends, which is a pattern observed in other large plant genomes [34, 35]. Defense gene density (blue track) is similar to overall gene density (orange track). Most significant sex-associated markers are located on the third largest scaffold (chromosome X; Scaffold_1533), which suggests that this scaffold is the putative X chromosome. We identified the set of sex-associated markers using mapping population USDA 2017014 based on 281 offspring and two parental genotypes (see Supporting Methods and Supplementary Data Table S8).

### Analysis of molecular evolution in syntenic gene blocks

Syntenic gene blocks are depicted in the center track of the circos plot, with most syntenic blocks occurring within the same scaffold (Figure 2D). To identify syntenic blocks, we used MCScanX. The resulting collinearity file from MCScanx is provided as a supplementary file (Supplementary Data Table S17).

A depiction of an intra-hop syntenic block on chromosome 5 (Scaffold_172) shows a proximal arrangement of disease resistance-associated genes, along with alpha-humulene synthase (Figure 2E). Figure 3A shows syntenic blocks shared between the largest 10 scaffolds in the hop and hemp genomes, highlighting extensive sequence similarity shared between hop and hemp. We also observe large regions of genomic sequence that are unique to hop chromosomes, particularly in chromosomes 3, 4, 6, and 9, that lack syntenic gene blocks and potentially correspond to centromeric regions.

Figures 3B and 3C show two syntenic blocks shared between hop and hemp. The syntenic block in Figure 3B contains disease response-associated genes and a copy of alpha-humulene synthase. Figure 3C shows genes associated with the cannabinoid synthase pathway, including cannabidiolic acid synthase (CBDAS), CBDAS-like 1, as well as disease response-associated genes.

**Figure 4:**
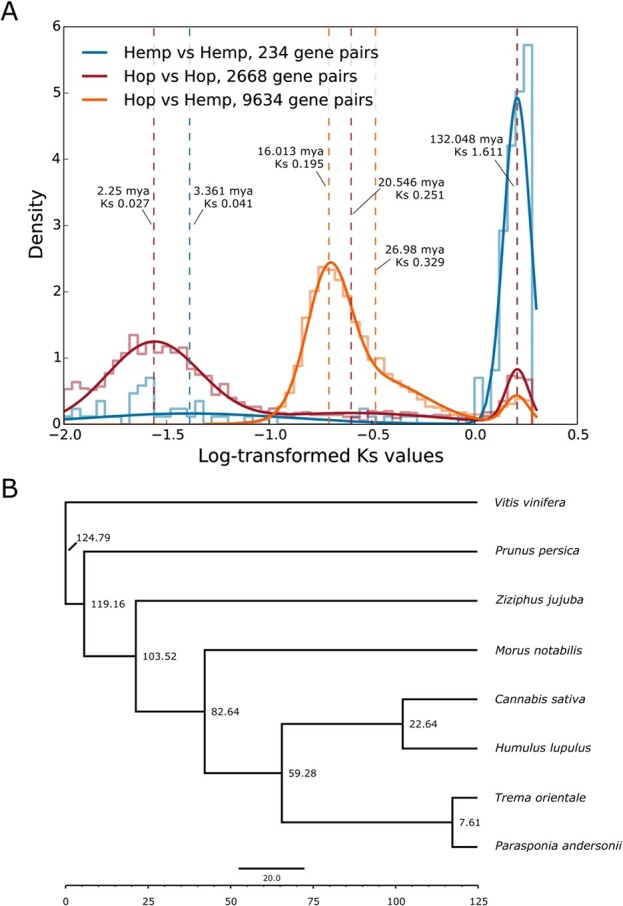
Estimates of molecular evolution and divergence time. **A)** Distribution of log-transformed Ks values from collinear gene pairs. The mixture model is superimposed over the histogram, and dashed lines correspond to the location of means identified by the mixture model. Time is included for each dashed line and is calculated using the equation T = Ks/2*λ* [36] and the substitution rate (λ) of 6.1 × 10^−9^. **B)** Bayesian time tree providing estimated dates of divergence between species. The dates are estimated by MCMCTree, incorporating fossil data and the species tree from OrthoFinder.

All putative copies of CBDAS or CBDAS-like genes in hop are present on a single scaffold (chromosome 9; Scaffold_191), the tenth largest scaffold in the assembly (HUMLU_CAS0069948.t1.p1 [1376 743:1378473], HUMLU_CAS0071060.t1.p1 [23265900:23268440], HUMLU_CAS0071138.t1.p1 [25580810:25581350], HUMLU_CAS0071292.t1.p1 [31 796 694:31798322], HUMLU_CAS0071300.t1.p1 [32 026 872:32028500], HUMLU_CAS0071316.t1.p1 [32 700 940:32709515], HUMLU_CAS0071321.t1.p1 [32 884 586:32886312], HUMLU_CAS0071330.t1.p1 [33088449,33091968]). Only one copy is expressed in hop and is located distantly from the other copies. The remaining copies can be grouped into approximately two locations. Three of these genes (HUMLU_CAS0071060.t1.p1, HUMLU_CAS0071138.t1.p1, HUMLU_CAS0071292.t1.p1) belong to syntenic blocks, with the latter two occurring in the same syntenic block.

On a genome-wide scale, syntenic blocks in hop have expanded LTR content, with LTRs distributed between anchor and non-anchor genes (Supplementary Data Figure S7A). The distance between anchor genes in hop is larger than in hemp (Supplementary Data Figure S7B), and syntenic blocks containing more genes typically have a smaller Ks (synonymous substitution rate) value, corresponding to more recent large-scale duplication events (Supplementary Data Figure S7C). Among syntenic blocks within hop, and between hop and hemp, enriched GO terms with the greatest statistical significance are associated with energy, metabolism, and development (Supplementary Data Figures S8 and S9).

We calculated the expected and observed rates of occurrence of syntenic blocks with both terpene and defense genes and found an enriched occurrence of terpene and defense genes that co-localize in the same syntenic block. While we expect to observe 7.8 syntenic blocks with both terpene and defense genes in hop vs hop blocks, we observe 11 blocks. We also expect to observe 16.9 blocks with both terpene and defense genes, but we observe 27 blocks with both terpene and defense genes (Supplementary Data Table S18). See also Supplementary Data Table S19 and Supplementary Data Table S20 for a list of genes with defense and terpene-associated GO terms in the 10 largest scaffolds, respectively.

We also assessed the total number of defense and terpene genes in syntenic blocks from each of the 10 largest scaffolds (Supplementary Data Figure S10), showing that chromosomes 8 and 5 (Scaffold_49 and Scaffold_172) have the most copies of defense genes in syntenic blocks in hop vs hop and hop vs hemp, respectively. Chromosome 8 (Scaffold_49) has significantly more defense genes than other scaffolds in the hop vs hemp blocks, with 172 defense genes, compared to the scaffold with the next highest abundance, at 129 defense genes (chromosome 3; Scaffold_76). Based on these results, chromosome 8 would be a useful target scaffold for further investigation of defense genes.

### Analysis of molecular evolution using Ks distributions

The optimal number of mixture model components for Ks from hop vs hop anchor genes was three, for hemp vs hemp was two, and for hop vs hemp was three. We interpreted the component mean nearest to the modal peak as the primary putative duplication event, corresponding to whole genome duplication or the speciation event between hop and hemp. For hop vs hop, the component means occur at Ks values of 0.027, 0.251, and 1.616; for hemp vs hemp at Ks values of 0.041 and 1.612; and for hop vs hemp at Ks values of 0.195, 0.329, and 1.605 (Figure 4A).

The hop vs hop peak occurring at Ks = 0.027 is the most recent large-scale duplication event in hop. The broad and diffuse peak area suggests duplications originating via different mechanisms and at different times, including both smaller duplication events and larger segmental duplications, as well as the speciation event marking the emergence of *Humulus lupulus* and *Humulus japonicus*.

For hop vs hemp, the peak occurring at Ks = 0.195 putatively marks the primary speciation event. The component mean occurring at Ks = 0.329 appears to overlap with the putative primary duplication event at Ks = 0.195. We do not necessarily interpret the component mean at Ks = 0.329 as a distinct duplication event. The primary putative duplication event shows a positive skew, characteristic of a trend described previously, wherein overfitting in the heavy right tail of the main peak can occur, leading to erroneous detection of duplication events [37]. We calculated a divergence date for hop and hemp of 16.013 mya, based on Ks = 0.195, indicating that this λ is concordant with the results of our Bayesian time tree (Figure 4A).

### Fossil-calibrated time tree

We computed a fossil-calibrated time tree based on single-copy orthologs present in eight species that we identified with OrthoFinder version 2.5.2 [33], including *Cannabis sativa*, *H. lupulus*, *Morus notabilis*, *Parasponia andersonii*, *Prunus persica*, *Trema orientale*, *Vitis vinifera*, and *Ziziphus jujuba.* We created the time tree using a concatenated multiple sequence alignment of the third codon position from four-fold degenerate codon sites (see Methods and Supporting Information), as well as a species tree from OrthoFinder, and fossil calibration dates. The orthologous gene groups are available on http://hopbase.cqls.oregonstate.edu/Downloads.php.

Based on a larger log-likelihood value, we determined that the independent log-normally distributed relaxed-clock model (clock = 2; clock2) [38] out-performed the strict molecular clock (clock = 1; clock1). The estimated time divergence for *Humulus* and *Cannabis* with the independent log-normally distributed relaxed-clock model is 22.6438 mya (95% highest posterior density [HPD] = 15.6728, 28.7994) (Figure 4B). The time trees generated using r8s and treePL are consistent with the estimated time of divergence based on the Ks analysis of collinear gene pairs from syntenic blocks (16.013 mya). The estimated date is 16.1843 mya (95% highest posterior density [HPD] = 5, 18.0982) from r8s (Supplementary Data Figure S12A) and 17.1096 from treePL (95% highest posterior density [HPD] = 17.0236, 17.2051) (Supplementary Data Figure S12B).

### Identification of orthologous genes and gene family analysis

**Figure 5:**
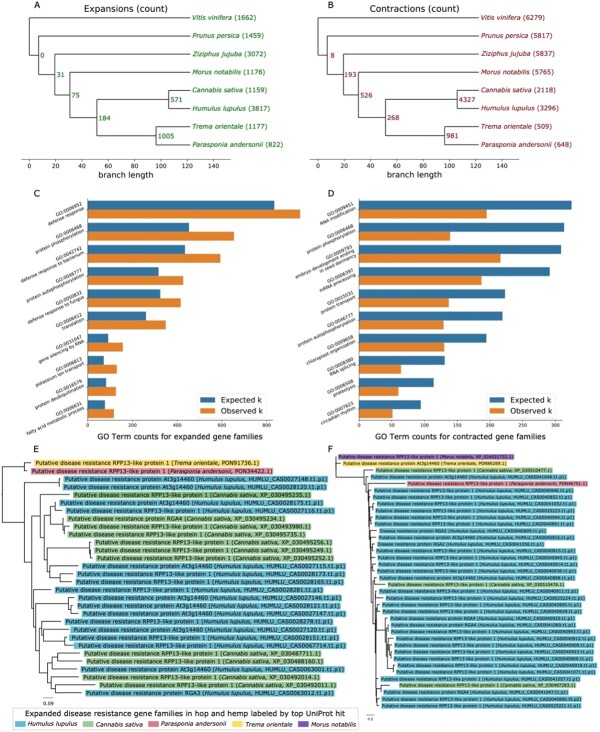
Gene family expansion and contraction. **A)** Tree showing the number of expanded gene families. **B)** Tree showing the number of contracted gene families. **C)** Bar chart showing statistically significant Biological Processes GO terms with the largest number of observed occurrences from expanded families identified with a hypergeometric test. **D)** Bar chart showing statistically significant Biological Processes GO terms with the largest number of observed occurrences from contracted families identified with a hypergeometric test. **E)** Gene family tree containing genes with significant similarity to putative disease resistance genes that are specific to the Cannabaceae family. Each branch is color-coded according to putative functional association. **F)** Gene family tree containing genes with significant similarity to putative disease resistance genes that are specific to the Cannabaceae family, except for one gene that occurs in mulberry (*Morus notabilis*). Each branch is color-coded according to putative functional association.

We identified contracted and expanded hop gene families (Figures 5A and 5B) and investigated functionally enriched GO terms in expanded and contracted groups (Figures 5C and 5D). There are 571 gene families expanded in both hop and hemp; 3817 gene families are expanded in hop only and 1159 gene families are expanded in hemp (Figure 5A). Among contracted gene families, 4327 gene families are shared between hop and hemp, with 3296 families specific to hop and 2118 families specific to hemp (Figure 5B).

Among the most significant functionally enriched Biological Processes GO terms in the expanded gene families are protein phosphorylation (GO:0006468), defense response (GO:0006952, GO:0042742, GO:0050832), and gene silencing by RNA (GO:0031047) (Figure 5C). Among contracted gene families, we find significant functionally enriched Biological. Processes GO terms including RNA modification (GO:0009451), protein phosphorylation (GO:0006468), embryo development ending in seed dormancy (GO:0009793), and circadian rhythm (GO:0007623) (Figure 5D). We further highlight two gene families associated with Biological Processes GO term “defense response” containing putative disease resistance genes that are expanded in hop (Figures 5E and 5F) and are also mostly restricted to the Cannabaceae.

### Orthologous gene groups containing genes associated with terpene and cannabinoid biosynthesis

**Figure 6:**
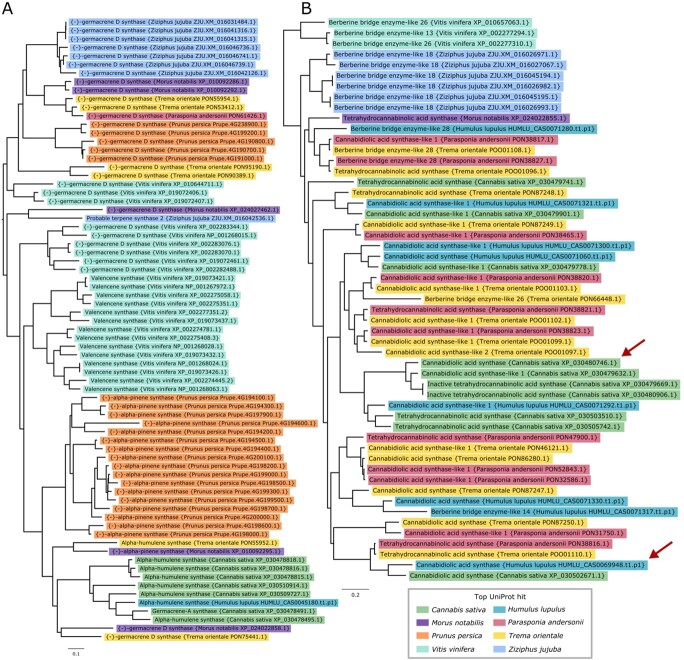
Gene family trees associated with terpene and cannabinoid biosynthesis. Phylogenetic trees demonstrate gene family expansions that are specific to hop and hemp and the Cannabaceae family. **A)** Gene family tree containing terpene synthase genes that is color-coded according to putative functional association. This tree shows species-specific divergence and specialization of terpene synthases in hop and hemp. Terpene synthases cluster by both function and species. **B)** Gene family tree containing genes putatively associated with cannabinoid synthase genes or Berberine bridge enzyme-like genes. Each branch is color-coded according to putative functional association. The red arrows point to the expressed copies of putative cannabidiolic acid synthase (CBDAS) in hop and CBDAS in hemp. This tree shows specific divergence of putative cannabinoid synthase genes in the Cannabaceae, derived from ancestral Berberine bridge enzyme-like proteins.

We highlight two phylogenetic trees in Figure 6 featuring secondary metabolic pathways of interest in hop. Figure 6A depicts a tree containing genes with similarity to terpene synthases, including germacrene A and D synthases, valencene synthase, alpha-pinene synthase, alpha-humulene synthase, and probable terpene synthase 2. Each of the different terpene synthases are grouped primarily according to species, suggesting that each of the different species has evolved distinct paralogous sets of terpene synthases. The topology of the tree suggests that each of these duplications occurred after speciation for the plants considered. Hop has one copy of alpha-humulene synthase in the tree, grouping closest to the six copies of alpha-humulene synthase and one copy of germacrene A synthase from *C. sativa*. Experimental validation is needed to confirm the functional activity of alpha-humulene synthase, as sequence similarity is not sufficient to assign function.

Figure 6B shows the orthologous gene group containing CBDAS, CBDAS-like 1 and 2, tetrahydrocannabinolic acid synthase (THCAS), inactive THCAS, and berberine bridge enzyme-like genes. A BBE-like gene is hypothesized to be the ancestor of the cannabinoid synthase genes following duplication and neofunctionalization [39]. The full length CDS in hop (HUMLU_CAS0069948.t1.p1) with similarity to CBDAS is expressed and shares closest similarity to a gene in *Cannabis* (XP_030502671.1). Although this *Cannabis* gene is annotated as CBDAS in our sequence similarity-based annotation, it is annotated as a CBDAS-like gene in the annotation of the hemp CBDRx assembly [30]. The only expressed copy of CBDAS in hemp (XP_030480746.1) is present in the gene tree in Figure 6B, clustering near copies of CBDAS-like 1, inactive THCAS, and a copy of CBDAS-like 1 in hop (HUMLU_CAS0071292.t1.p1).

The expressed copies of putative CBDAS in hop and CBDAS in hemp do not cluster together and are each related to other copies of putative cannabinoid synthase genes from *T. orientale* and *P. andersonii*. Further, both CBDAS and THCAS can produce small amounts of other cannabinoids [40]; therefore, further validation is required to identify the primary products that result from these synthases, especially for the expressed copy in hop.

## Discussion

### Genome assembly analysis

We have presented a chromosome-level assembly of Cascade that contains 3.47 Gb of genomic sequence in the largest 10 scaffolds, which we expect corresponds to the 10 chromosomes. The haploid size of the hop genome was previously estimated to be between 2.57 Gb [26] and 2.989 Gb [140] (Supplementary Data Table S5), and our k-mer-based estimate of the size of the Cascade hop genome is closer to the size of the assembly at 3.058 Gb. The size of the Dovetail assembly is larger than the estimated genome size by flow cytometry; however, the reported genome sizes in the literature demonstrate variation in genome size across cultivars. Variation in genome size is known to occur within plants of the same species [41] as a result of large structural variants [42].

Cascade has a highly heterozygous genome, which can be desirable in the cultivation of new varieties [10]. Using short-read DNA sequencing, we estimated the heterozygosity of the hop genome to be approximately 5%, which is similar to the range of other heterozygous genomes, including potato (4.8%) [43], *Vitis vinifera* cultivar Börner (3.1%) [44], *V. vinifera* cultivar PN40024 (7%) [45], and sunflower (10%) [46]. Heterozygosity does present challenges in assembly and annotation related to distinguishing between haplotype and paralogous sequences, especially in the case of recent duplications. Although we performed phasing and further efforts to detect haplotype contigs in the PacBio long-read assembly that provided the basis for the Dovetail assembly, heterozygosity continues to be a challenge for future work to overcome [27]. Hi-C construction of scaffolds does appear to reduce the inclusion of homologous primary contigs representing the corresponding haplotype. Further restricting BUSCO analysis to the largest 10 scaffolds reduced the number of duplicated BUSCOs (7.7% to 3.4%) while increasing the number of single-copy BUSCOs (88.2% to 92.6%), supporting the inclusion of the 10 scaffolds for downstream analyses that are sensitive to duplication. Polishing of the assembly using short-read Illumina sequencing further improved the quality of the scaffold sequences, based on the recovery of BUSCO genes.

Zhang *et al.* showed obvious segregation distortions in genetic mapping of hop, presumably caused by multivalent formation during meiosis [47]. These segregation distortions could ultimately influence marker positioning on linkage groups with the consequence of large-scale marker misplacement on genetic maps. The genetic map developed for our mapping population, USDA 2017014, was based upon SNP markers identified by genotyping-by-sequencing (GBS) of 281 offspring. Comparisons between the physical position of SNPs and the genetic map (Supplementary Data Figure S5) show marked divergences between the physical and genetic positions. Our results demonstrate the potential problems posed by using genetic maps to assemble contigs into chromosomal-scale scaffolds. Long-range interaction-based methods such as Dovetail Hi-C, coupled with PacBio long-read sequencing, allow for the assembly of contigs into chromosomal-scale scaffolds independently of genetic maps. Based upon our results it is recommended that future hop genome assemblies avoid use of genetic maps for scaffolding of large contigs and instead use methods such as long-range scaffolding with Hi-C or more traditional large insert bacterial artificial chromosome (BAC) libraries that overlap genomic regions.

Our new estimate of repeat content of the Dovetail assembly for Cascade, at 64.25%, is similar to previous estimates for repeat content, with repeat content for *Humulus lupulus* Japanese wild hops at 60.1%, var. *lupulus* at 61.3%, and var. *cordifolius* at 59.2% [48]. Previously, in the PacBio long-read assembly, we estimated the total repeat content for the assembly at 71.46% [27]. Assembly with Hi-Rise did result in 1027 breakage points to the PacBio assembly, as well as 8131 joins, and it is possible that these breakages resulted in a shuffling of genome content that changed the resulting total repeat percentage. An earlier estimate of repeat content for *H. lupulus* cultivar Shinshu Wase was 34.7% [26]. However, assemblies generated with short-read sequencing likely underestimate repeat content by not comprehensively capturing intergenic and repeat regions. The repeat content of the closely related *C. sativa* genome is 64% [48]. Although we observe syntenic blocks expanded in hop due to LTR insertion, on a genome-wide scale, the similarity in repeat content between hop and hemp suggests that LTRs do not contribute to larger genome size in hop.

### Genomic content of syntenic blocks

The syntenic blocks in Figures 3B and 3C show an expansion of LTR sequences in hop, and we observe on a genome-wide scale that syntenic blocks in hop have expanded LTR content (Supplementary Data Figure S7A). LTRs impact gene expression by altering the spacing and organization of accessible chromatin regions (ACRs), which can be involved in regulation of gene expression by harboring accessible *cis*-regulatory elements (CREs), including transcription factor binding sites [49]. In maize, single-nucleotide polymorphisms in ACRs are responsible for ~40% of heritable variance in quantitative traits, highlighting the importance of identifying ACRs containing regulatory DNA [50]. For hop, an open question is whether the apparent expansion of LTR content in syntenic blocks has influenced the evolution and regulation of genes involved in traits of interest. Future work will be necessary to identify CREs, ACRs, and assess their role in controlling gene expression [51].

In syntenic gene blocks of hop and hemp, we observe multiple copies of defense response-associated genes that are co-localized with terpene synthase genes (Figures 2E and 3B). Given the rate of occurrence of these syntenic blocks with each individual gene family, we expect to observe 7.8 syntenic blocks with both terpene and defense genes in hop vs hop blocks, but we observe 11 blocks. In hop vs hemp, we expect to observe 16.9 blocks with both terpene and defense genes; however, we observe 27 blocks with both terpene and defense genes (Supplementary Data Table S18). Further, the 11 syntenic blocks from hop vs hop with a terpene gene also have a defense gene. In hop vs hemp blocks, 27 out of the 32 blocks that have a terpene gene also have a defense gene, highlighting the dual role of terpene synthases. The presence of these syntenic gene blocks with both terpene and defense genes in hop and hemp also points to the importance of these genes in the common ancestor.

Terpenes contribute to chemical response to pathogens and herbivores [52]. The evolution of genes involved in specialized plant metabolism reflects a response to dynamic environmental pressures. Specialized metabolic genes can be arranged in clusters, emerging via duplication [52]. In particular, unequal crossing-over during meiosis can cause tandem duplication, giving rise to a second gene located nearby [53, 54]. Duplication by transposon mobilization can create a large distance between two gene copies.

In plants, resistance genes arise predominantly via tandem and segmental duplications [55]. In barley, duplication of resistance genes was caused disproportionately by tandem duplication [56]. Hop forms multivalents during meiosis, which is the pairing of non-homologous chromosomes, potentially resulting in tandem gene duplicates [47, 57]. The multiple copies of defense response-associated genes may have evolved as a result of duplication.

Given the potential for pathogens to overcome plant disease resistance, further investigation is necessary to understand the genomic features that contribute to disease resistance in hop. An improved understanding of the genomic organization of disease response-associated genes may guide identification of biomarkers and assist in breeding efforts to develop cultivars with enhanced disease-resistance.

### Gene family expansion and contraction

Among the most enriched GO terms in both expanded and contracted gene families, the GO term “protein phosphorylation” (GO:0006468) occurs as a significant Biological Processes GO term for both contracted and expanded gene families. Hop genes with similarity to UniProt genes associated with protein phosphorylation include Disease resistance protein RPP8 (Q8W4J9), chloroplastic Geranylgeranyl diphosphate reductase (Q9ZS34), Protein PHYTOCHROME-DEPENDENT LATE-FLOWERING (F4IDB2), Ultraviolet-B receptor UVR8 (Q9FN03), LEAF RUST 10 DISEASE-RESISTANCE LOCUS RECEPTOR-LIKE PROTEIN KINASE-like 1.2 (P0C5E2), and Two-component response regulator-like APRR1 (Q9LKL2), which is involved in light-mediated flowering response [58]. Although we observe genes with the same putative function occurring in both expanded and contracted gene families, in our analysis these genes cluster into different orthologous groups, suggesting genes with these putative functions have undergone duplication and sub-functionalization [53].

The trees in Figures 5E and 5F contain disease response-associated genes present in the expanded gene families. Both gene trees contain multiple copies of RPP13-like protein 1, which is associated with resistance to downy mildew in *Arabidopsis* and is characterized by high amino acid divergence within the functional domain [59]. Resistance gene analogs (RGA2, RGA3, RGA4) are also present in the two trees [60].

Overall, we found that defense response genes are enriched in gene families and syntenic gene blocks and tend to co-localize with important metabolic terpene-related genes. Defense and terpene genes also share a similar density distribution, as shown in the circos plot (Figure 2D). Further, we found that chromosomes 8 and 5 (Scaffold_49 and Scaffold_172) have the most abundant occurrence of defense genes in syntenic blocks in hop vs hop and hop vs hemp, respectively, and contain terpene synthase genes. Defense response is a dynamic and continual challenge for cultivating hop. We hope our identification and analyses of these loci will guide future research efforts.

### Date of species divergence

We estimate the divergence date for hop and hemp to be approximately 16.013 mya using Ks (Figure 4A) and approximately 22.64 mya with the Bayesian inference-based time tree. Our new estimates of time divergence for hop and hemp approximately agree with previous estimates between 18.23 and 27.8 mya [16, 18, 61–65]. Each of these estimates is accompanied by its own uncertainty interval, increasing the overall range of divergence time. Our specific point estimates for the divergence time fall within a narrower and slightly more-recent window than previous estimates; however, if we consider the HPD interval of 15.6728 to 28.7994 from the Bayesian time tree, our results are consistent with previous estimates.

The time equation, $T= Ks/2\lambda$, is dependent on the value of λ, and there are different estimates of λ among plants, including 6.1 × 10^−9^ [66] and 1.5 × 10^−8^ [47] for *Arabidopsis*, and a range of 2.1 × 10^−9^ to 2.9 × 10^−9^ for the monocot-dicot divergence [67]. If we apply other values of λ, including λ = 2.1 × 10^−9^ [67, 68], we calculate a divergence date of 46.43 mya. With λ = 1.23 × 10^−9^ [69], we calculate a divergence date of 79.26 mya, which is closer to the crown age of Cannabaceae, estimated to be 87.4 mya [18]. Our estimated divergence date using λ = 6.1 × 10^−9^ agrees with our date estimated by Bayesian inference, and has previously been applied to closely related species, hemp and mulberry [15], suggesting that it is reflective of the rate of evolution in hop.

The overall sparsity of the fossil record, as well as changes to the assignment of fossils, speaks to the uncertainty of the results. Multiple fossils from extinct *Humulus* species are dated to the Oligocene (23.03–33.9 mya) [16]. Older, more debatable fossil evidence for *Humulus* includes a leaf fossil, with a date of 34.07 mya based on ^40^Ar/^39^Ar radiometric dating [16, 70], from Florissant, Colorado, USA [71]. However, this older fossil remains questionable because it was originally identified as *Vitis* [71], and subsequently re-assigned to *Humulus* [72]. Collinson estimated the time of origin for *Humulus* and the extinct genus *Humularia* at the boundary of the Eocene and Oligocene epochs, corresponding to 33.9 mya [73]; however, this date hinges on the reliability of the Florissant leaf fossil, which McPartland notes was insufficient evidence to warrant conclusive assignment to either family based on the lack of diagnostic fruit [16, 74]. Based on the uncertainty related to placing this fossil, we opted not to incorporate it into our fossil calibration.

## Conclusion and future work

Our chromosome-level genome assembly of Cascade lays the foundation for further evolutionary and biodiversity studies focusing on *Humulus* and the Cannabaceae. This genomic resource will provide a better understanding of content and organization of genes involved in flowering time [75], growth and development, defense response, and metabolism. Future work is needed to identify and map centromeres and telomeres of the hop genome, and to resolve biodiversity and structural variation across *H. lupulus* cultivars.

## Materials and methods

### Genome sequencing and assembly

Hi-C libraries were used to scaffold and correct the contigs from the PacBio long-read assembly [27] using HiRise [76] by Dovetail Genomics. DNA was extracted from Cascade leaves using a method previously described [27], which involved a modified CTAB method to reduce the inclusion of small, sheared DNA fragments. Dovetail scaffolds were polished with a set of 563 456 691 DNA short reads from Cascade using the Polca polishing tool [77]. The paired DNA reads were 150 bp in length. We used samtools-1.11 flagstat to assess the mapping rate of the DNA short reads to the Dovetail assembly. We used BUSCO v5.2.2 [78] to assess the assembly completeness, which also incorporated Augustus-3.3.2 [79] and both Embryophyta and Viridiplantae databases from OrthoDB v10 [80].

### Repeat annotation

We identified repeat sequences using an approach described previously [27]. Briefly, we developed a *de novo* set of long terminal retrotransposon (LTR) annotations using suffixerator (GenomeTools) 1.6.1 [81], LTRharvest (GenomeTools) 1.6.1 [82], LTR_FINDER_parallel v1.1 [83], and LTR_retriever v2.7 [84]. We used suffixerator to index the assembly, then LTRharvest and LTR_FINDER_parallel were used to identify LTRs, and finally, LTR_retriever was used to synthesize and refine the results of LTRharvest and LTR_FINDER_parallel. We identified non-LTR types of repeats from plants using a database from MIPS PlantsDB [85]. The *de novo* set of LTRs and the database of plant repeats were aligned to the assembly using RepeatMasker version 4.1.0 [86]. The repeat annotation pipeline was performed on each scaffold separately. The detailed pipeline for repeat annotation can be found at RepeatAnnotationPipeline.md on GitHub.

### Gene model development

We developed our set of gene models with Transdecoder-v5.5.0 [87] and MAKER [88, 89]. In cases of overlapping gene models, we assigned preference to the Transdecoder gene models, because Transdecoder directly incorporates transcript expression evidence into gene model development. We incorporated RNA-seq data from lupulin glands and leaf (see methods in Supporting Information [SI]); leaf, meristem, and stem tissues [27], as well as from hop cones during critical developmental stages [90]. Putative gene functions were assigned based on similarity to known proteins from UniProt (accessed 08/24/2020 using search term: taxonomy:"Embryophyta [3193]” AND reviewed:yes) [91] and Pfam protein domains (Pfam release 33.1) [92]. The detailed pipeline for generating the gene models and then assigning putative function is provided in SI methods (Supplementary Data Table S14 and Supplementary Data Table S15) and on the GitHub project page in the directory ‘GeneModels.’

### Synonymous substitution rate (Ks)

To assess molecular evolution in hop and hemp, we calculated the synonymous substitution rate (Ks) [93–95] for anchor gene pairs identified with MCScanX [54]. Synonymous substitutions do not change the encoded amino acid [96], and because substitution occurs at an approximately constant rate, the substitution rate can be treated as a proxy for elapsed time since the duplication of paralogous genes [97, 98].

We visualized syntenic blocks on a genome-wide scale with SynVisio [99], requiring a minimum MATCH_SIZE of nine, corresponding to at least 10 anchor genes per block. We also ran MCScanX with default settings for downstream analysis and visualized the blocks with Integrative Genomics Viewer (IGV) [100].

We performed a codon alignment for each anchor gene pair using MACSE alignSequences and exportAlignment [101]. We calculated Ks for each collinear gene pair individually [102] using the yn00 package within CodeML [103, 104]. We assessed functional enrichment in the syntenic blocks of hop vs hop and hop vs hemp with a hypergeometric test and performed a Benjamini-Hochberg multiple test correction [6] to obtain a q-value for each GO term ($FDR<0.05$).

### Analysis of molecular evolution using Ks distributions

Divergence dates are often estimated using Ks and the mutations per site per year [36], denoted as λ, with the formula $T= Ks/2\lambda$ [36], where $T$ is the length of time elapsed since the time of duplication or divergence [106]. A known divergence time is required to determine λ, which is reliant upon evidence from the fossil record [67]. There are different estimates of λ for plants, including 6.1 × 10^−9^ [66] and 1.5 × 10^−8^ [36] for *Arabidopsis*. In a previous study, the divergence date between *H. lupulus* and *Humulus japonicus* was estimated to be 3.74 mya based on Ks=$0.0157\pm 0.0056$ using the *rbc*L sequence [68], along with λ = 2.1 × 10^−9^ [67]. In subsequent work, Murakami *et al.* used λ = 1.23 × 10^−9^ [69] to calculate the divergence date for European, North American, and Asian hop lineages, and provided an updated estimate of the divergence date between *H. lupulus* and *H. japonicus* as 6.38 mya [107]. We used λ = 6.1 × 10^−9^ [15, 66, 108], which was also used to calculate the divergence time between *Cannabis sativa* and *Morus notabilis* [15].

We restricted Ks values to ≥0.01 and ≤ 2.0 [98, 106], to limit the inclusion of Ks values from allelic variants [109, 110], and to avoid saturation at large Ks values [106, 111, 112]. Ks = 0.01 corresponds to the approximate Ks value marking the divergence of *H. lupulus* and *H. japonicus* [68].

We performed density estimation with log-transformed Ks values using Gaussian finite mixture modeling within the densityMclust function of mclust version 5.4.7 [113] in R version 4.0.3 [114]. Log-transformation allows better detection of peaks corresponding to duplication events [106]. We used the integrated complete-data likelihood (ICL) to select the number of clusters. ICL penalizes clusters that overlap excessively and tends to prefer clusters with well-delineated separation [115]. Our approach was adapted from previously described methods [106, 116–119].

### Fossil-calibrated time tree

We used OrthoFinder version 2.5.2 [33] to identify orthologous genes in eight species: *C. sativa*, *H. lupulus*, *Morus notabilis*, *Parasponia andersonii*, *Prunus persica*, *Trema orientale*, *Vitis vinifera*, and *Ziziphus jujuba*. We collected single-copy orthologs present in all species and aligned the sequences with MACSE alignSequences and exportAlignment. Then, we extracted the third codon position from four-fold degenerate codon sites and concatenated the third positions to create a single alignment.

We used MCMCTree to perform a Bayesian estimate of divergence times, incorporating the concatenated alignment, the species-level phylogenetic tree from OrthoFinder, and fossil calibration data. We evaluated two molecular clock models with MCMCTree: strict molecular clock and independent log-normally distributed relaxed-clock model (clock = 2) [38], using a likelihood-ratio test (LRT) to compare molecular clock models [120] with the equation $LRT=-2\Big(\mathit{\ln}\Big({L}_s\Big)-\mathit{\ln}\Big({L}_g\Big)\Big)$. MCMCTree parameters are described in Supplementary Data Methods and Supplementary Data Figure S12. To approach this problem from multiple angles and obtain a more confident result, we also used r8s [121] and treePL [122] to estimate the time of divergence (see Supplementary Data Methods and Supplementary Data Figure S13).

### Gene family expansion and contraction

We investigated the expansion and contraction of gene family size in a phylogenetic context with CAFE [123]. We incorporated our time tree, along with the size of each orthologous gene group from OrthoFinder. We required orthogroups to contain no more than 100 genes from a single taxon [124]. Trees were visualized with FigTree 1.4.4 [125].

We assessed GO term functional enrichment in expanded and contracted gene families in hop. We applied a hypergeometric test to identify enriched GO terms among all genes and all species that occurred in the expanded and contracted gene families. Then, we performed a Benjamini-Hochberg multiple test correction [105] to obtain a q-value for each GO term.

We collected the top functionally enriched GO terms among all statistically significant GO terms ($FDR<0.05$). We visualized the top GO terms with $FDR<1e-5$, sorting by observed $k$. Depicted in Figure 5C and 5D are a collection of these top 10 functionally enriched Biological Processes GO terms. GO term associations are available for download at http://hopbase.cqls.oregonstate.edu/Downloads.php.

### Construction of the linkage map

A controlled bi-parental mapping population was developed by crossing the female hop line “Comet” [126] by the male USDA germplasm accession 64 035M. Seeds were vernalized, germinated, and grown in the greenhouse as previously reported [127]. The population used for this study consisted of 281 offspring and the corresponding parents. DNA extraction for all genotypes, library preparation for Illumina-based genotyping-by-sequencing (GBS), and Illumina sequencing were all performed as previously reported [128].

Genetic mapping was performed as previously described [128]. Briefly, SNPtag data files were imported into Microsoft Excel and numerical values (0, 1, 2) were converted to A, H, B (representing AA, AB, BB). Segregation for each locus was obtained and tested for goodness-of-fit with predicted test-cross segregation (1:1) or F2-segregation (1:2:1) using chi-squared tests. Loci with significant chi-squared test statistics for goodness-of-fit for test-cross segregation (1:1) were combined with loci with significant test statistics for F2-segregation and subsequently combined into male and female data sets. These two data sets were imported as F2-formated files and separately analyzed in JoinMap 5.0 [129]. Genetic maps were estimated using both maximum likelihood (ML) and regression mapping (with two rounds of map estimation). Under ML mapping, loci with resulting high “NN Fit (cM)” (NN Fit values >100) were excluded from map estimation, and map estimation was re-run until no further high NN Fit values were observed. The resulting male and female F2 genetic maps were subsequently joined into a single “CP-Method” dataset and genotypes were re-coded with CP-Method codes (i.e.: ll, lm, nn, np, hh, hk and kk). Regression mapping in JoinMap 5.0 using CP-Methods (again, with two rounds of map estimation) was subsequently performed to obtain the overall unbiased length of the genetic map. ML mapping using CP-Method was also performed to provide a more-accurate placement of markers for genetic map estimation. The resulting genetic distance between markers using ML was always overestimated. As a result, the ML map marker distances were adjusted downwards to match the overall genetic map estimate obtained via use of regression mapping in JoinMap 5.0.

## Supplementary Material

Web_Material_uhac281

## Data Availability

The data that support the findings of this study are openly available on the Downloads page of http://hopbase.cqls.oregonstate.edu/ and under NCBI BioProject ID PRJNA562558. Analysis pipelines, scripts, and specific commands are included on the GitHub project page, at https://github.com/padgittl/CascadeDovetail.
