# Supplementary material for: An improved assembly of the “Cascade” hop (Humulus lupulus) genome uncovers signatures of molecular evolution and refines time of divergence estimates for the Cannabaceae family
Source: Hortic Res. 2022 Dec 7;10(2):uhac281. doi: 10.1093/hr/uhac281 (PMC9930403; doi:10.1093/hr/uhac281)
Supplement: Web_Material_uhac281 [file web_material_uhac281.zip › SupplementaryInformation_revision2_clean.docx]

## Supplemental Information for An improved chromosome-level assembly of the “Cascade” hop (*Humulus lupulus*) genome uncovers signatures of molecular evolution and refines time of divergence estimates for the Cannabaceae family

Running title: Cascade hop genome assembly

Lillian K Padgitt-Cobb^1^, Nicholi J Pitra^2^, Paul D Matthews^2^, John A Henning^3,4,^*, David A Hendrix^1,5,^*

padgittl@oregonstate.edu, npitra@hopsteiner.com, pmatthews@hopsteiner.com

* co-corresponding authors: david.hendrix@oregonstate.edu, john.henning@usda.gov

1. Department of Biochemistry and Biophysics, Oregon State University, Corvallis, Oregon

2. Department of Research and Development, Hopsteiner, S.S. Steiner, Inc., 1 West Washington Avenue, Yakima, Washington 98903, United States

3. Forage Seed and Cereal Research Unit, USDA-ARS, Corvallis, Oregon

4. Department of Crop and Soil Science, Oregon State University, Corvallis, Oregon

5. School of Electrical Engineering and Computer Science, Oregon State University, Corvallis, Oregon

## Supplemental Information

## The following Supplemental Information is available for this article:

**Fig. S1.** Distribution of Dovetail Hi-C read insert sizes

**Fig. S2.** Dovetail Hi-C link density histogram

**Fig. S3.** Plot showing contiguity of the input assembly and the final HiRise scaffolds

**Fig. S4.** Nucleotide, dinucleotide, and trinucleotide content of the assembly

**Fig. S5.** Genetic map vs physical map positions

**Fig. S6.** Percent identity to UniProt genes among Transdecoder and MAKER gene models

**Fig. S7.** Comparison of inter-anchor distances and average Ks values in hop and hemp syntenic blocks

**Fig. S8.** Functional enrichment of GO terms in hop vs hop syntenic blocks

**Fig. S9.** Functional enrichment of GO terms in hop vs hemp syntenic blocks

**Fig. S10.** Total number of defense and terpene genes in syntenic blocks

**Fig. S11.** OrthoFinder results

**Fig. S12.** MCMCTree convergence

**Fig. S13.** Estimation of time divergence using r8s and treePL

**Table S1.** Statistics about Hi-C libraries and HiRise assembly

**Table S2.** Analysis of polished assembly quality

**Table S3.** Comparative assembly statistics

**Table S4.** Assembly BUSCO results

**Table S5.** Estimated genome sizes of *Humulus* and closely related species

**Table S6.** Hop genome heterozygosity and repeat content based on short-read DNA sequencing

**Table S7.** Repeat percentages relative to total repeat content and assembly length

**Table S8.** Linkage group statistics for the mapping population USDA 2017014

**Table S9**. Sex-associated genetic map for mapping population USDA 2017014 (provided as a separate file)

**Table S10**. Sex-associated linkage disequilibrium map for mapping population USDA 2017014 (provided as a separate file)

**Table S11.** Transdecoder gene model results

**Table S12.** Gene model BUSCO results

**Table S13.** Gene statistics

**Table S14.** Pfam repeat-associated domains (this table is provided as a separate Excel file)

**Table S15.** Conditional repeat Pfam domains

**Table S16.** Number of genes with GO terms

**Table S17.** MCSCanX collinearity output file (provided as a separate file)

**Table S18.** Syntenic blocks with defense and terpene-associated genes in ten largest scaffolds

**Table S19.** Genes with defense-associated GO terms in ten largest scaffolds (provided as a separate file)

**Table S20.** Genes with terpene-associated GO terms in ten largest scaffolds (provided as a separate file)

**Methods S1** Detailed descriptions of SNP identification and filtering; genome size and heterozygosity; gene model development, quality assessment, and assignment of putative function; and molecular evolutionary analyses.

**Supplemental** **References**

**Fig. S1.** **Distribution of Dovetail Hi-C read insert sizes.** Scatter plot showing the distribution of insert sizes in the Dovetail library. The distance between the forward and reverse reads is given on the x-axis in base pairs, and the probability of observing a read pair with a given insert size is shown on the y-axis.


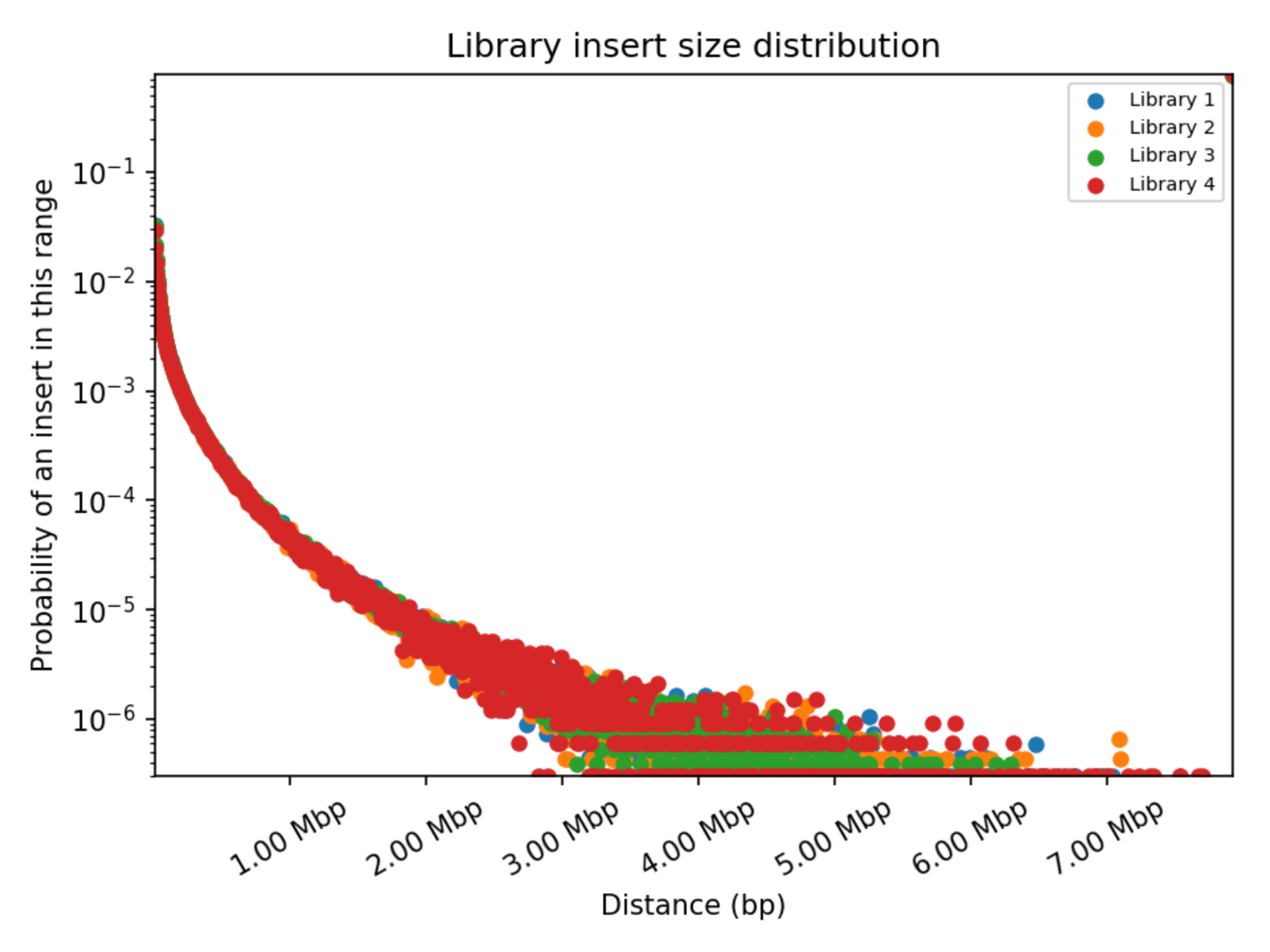


**Fig. S2. Dovetail Hi-C link density histogram.** This link density histogram shows the mapping positions of the first and second read in the read pair respectively, grouped into bins on the x- and y-axes. The color of each square gives the number of read pairs within that bin. White vertical and black horizontal lines have been added to show the borders between scaffolds. Scaffolds less than 1 Mb are excluded.


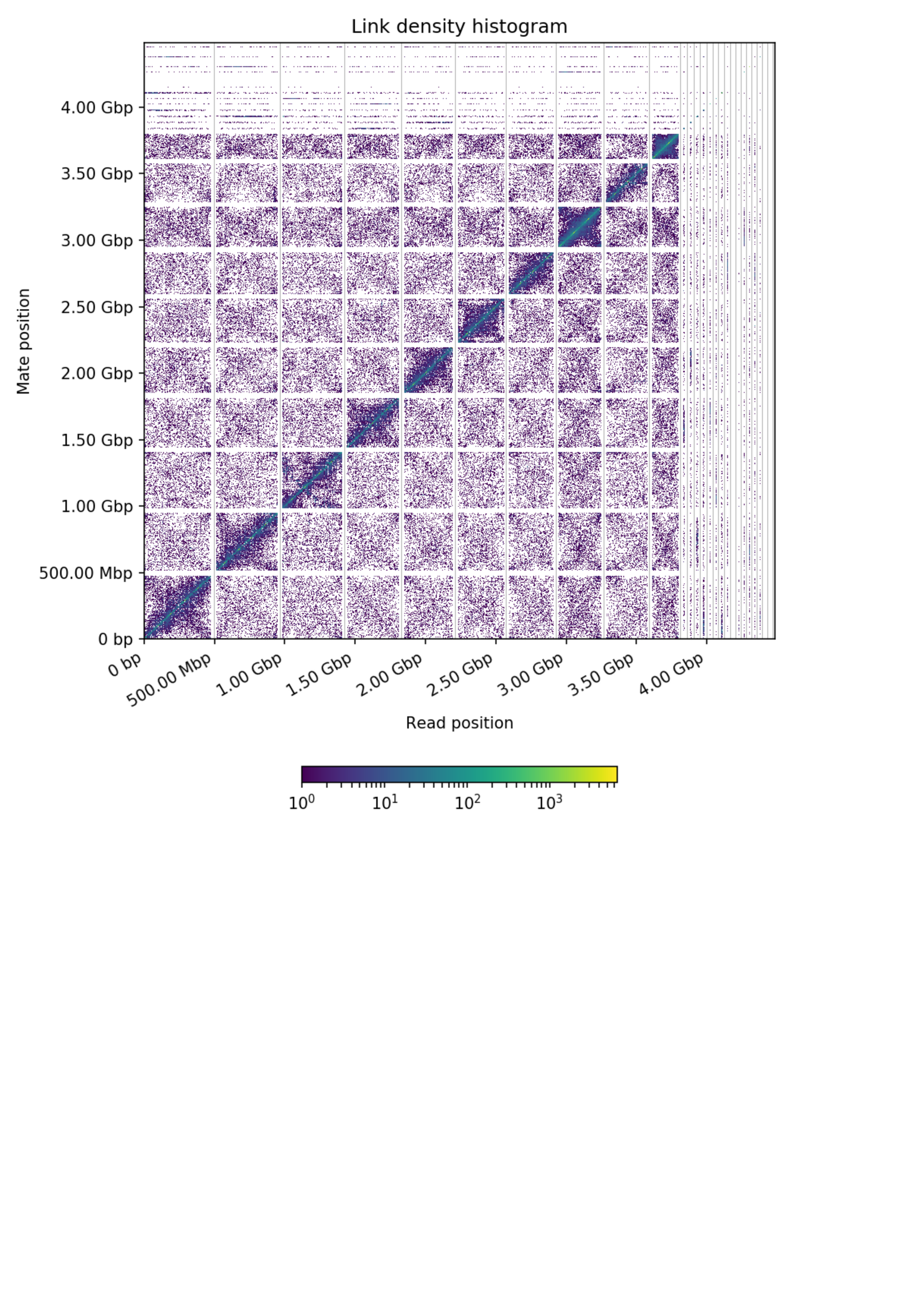


**Fig. S3. Plot showing contiguity of the input assembly and the final HiRise scaffolds.** This plot shows a comparison of the contiguity of the input assembly and the final HiRise scaffolds. Each curve shows the fraction of the total length of the assembly present in scaffolds of a given length or smaller. The fraction of the assembly is indicated on the y-axis and the scaffold length in base pairs is given on the x-axis. The two dashed lines mark the N50 and N90 lengths of each assembly.


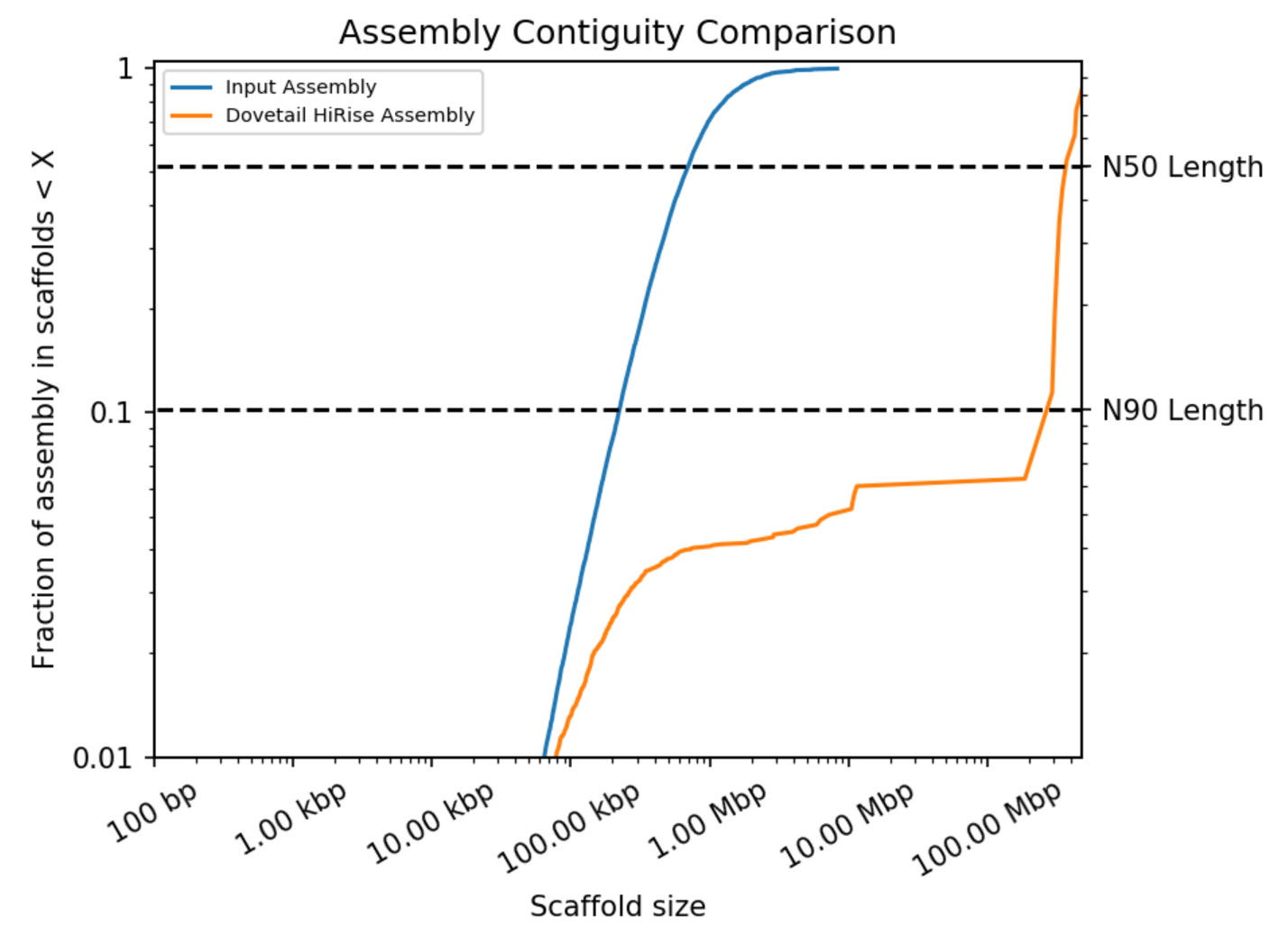


**Fig. S4. Nucleotide, dinucleotide, and trinucleotide content of the assembly. A)** A pie chart shows the percentages for each nucleotide in the largest ten scaffolds. **B)** Heatmap for the dinucleotide composition showing the enrichment of a given dinucleotide. The enrichment score is calculated by dividing the observed frequency of a dinucleotide by the expected frequency. The dinucleotide CG shows depletion, with an enrichment score of 0.66. **C)** Scatter plot showing the expected frequency of a trinucleotide on the x-axis along with the observed frequency on the y-axis. The trinucleotide CHH occurs more frequently than expected; CHH is associated with DNA methylation.
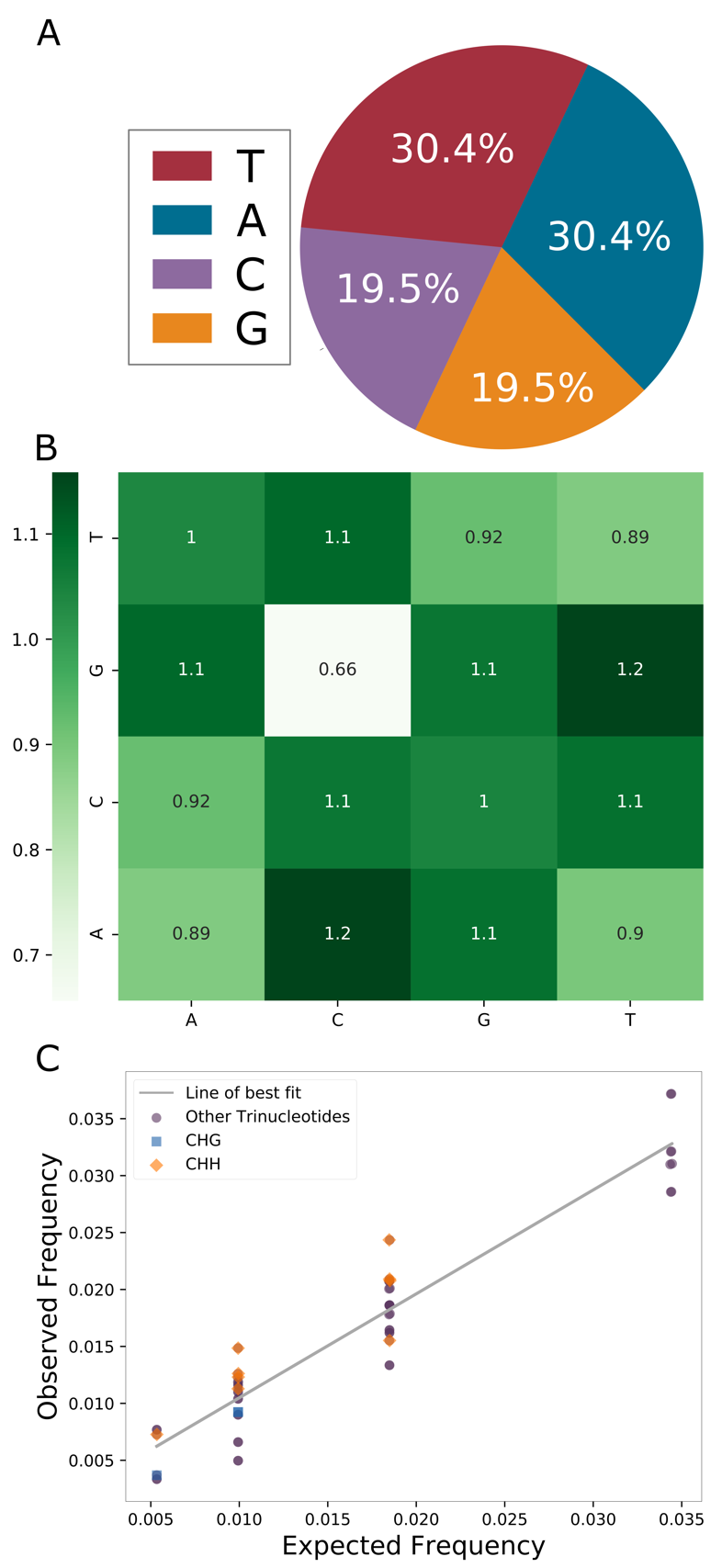


**Fig. S5. Physical map vs linkage disequilibrium map.** The subplots in this figure are the ten largest scaffolds and provide a comparison of physical vs genetic distance. The x-axis is the physical position of sex-associated markers along the length of the scaffold in megabases. The y-axis shows linkage disequilibrium units (LDU).


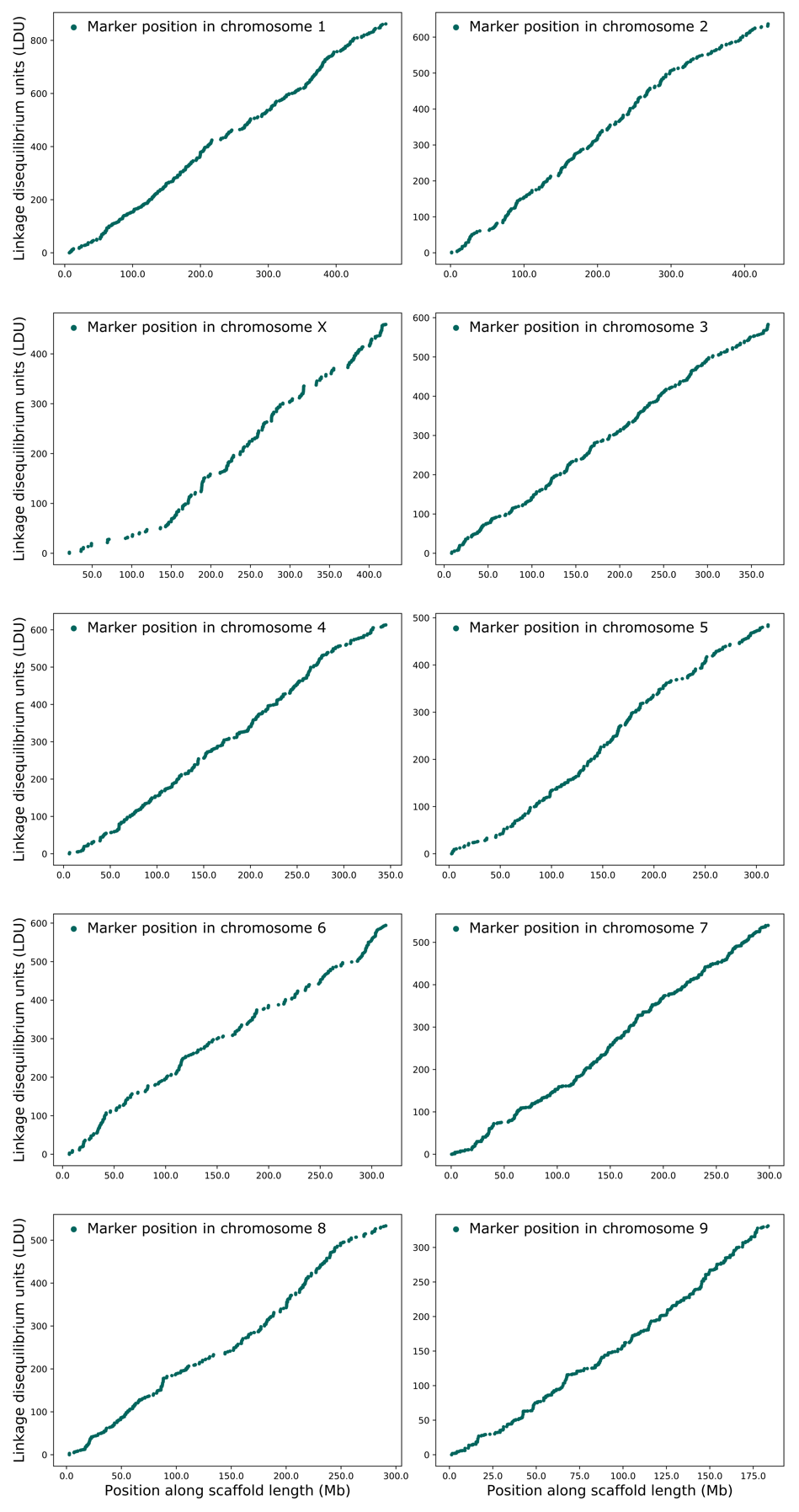


**Fig. S6. Percent identity to UniProt genes among Transdecoder and MAKER gene models.** Histogram of the percent identity to UniProt genes among the set of MAKER gene models and the set of Transdecoder gene models. Transdecoder gene models overall share greater similarity with known genes than MAKER gene models.


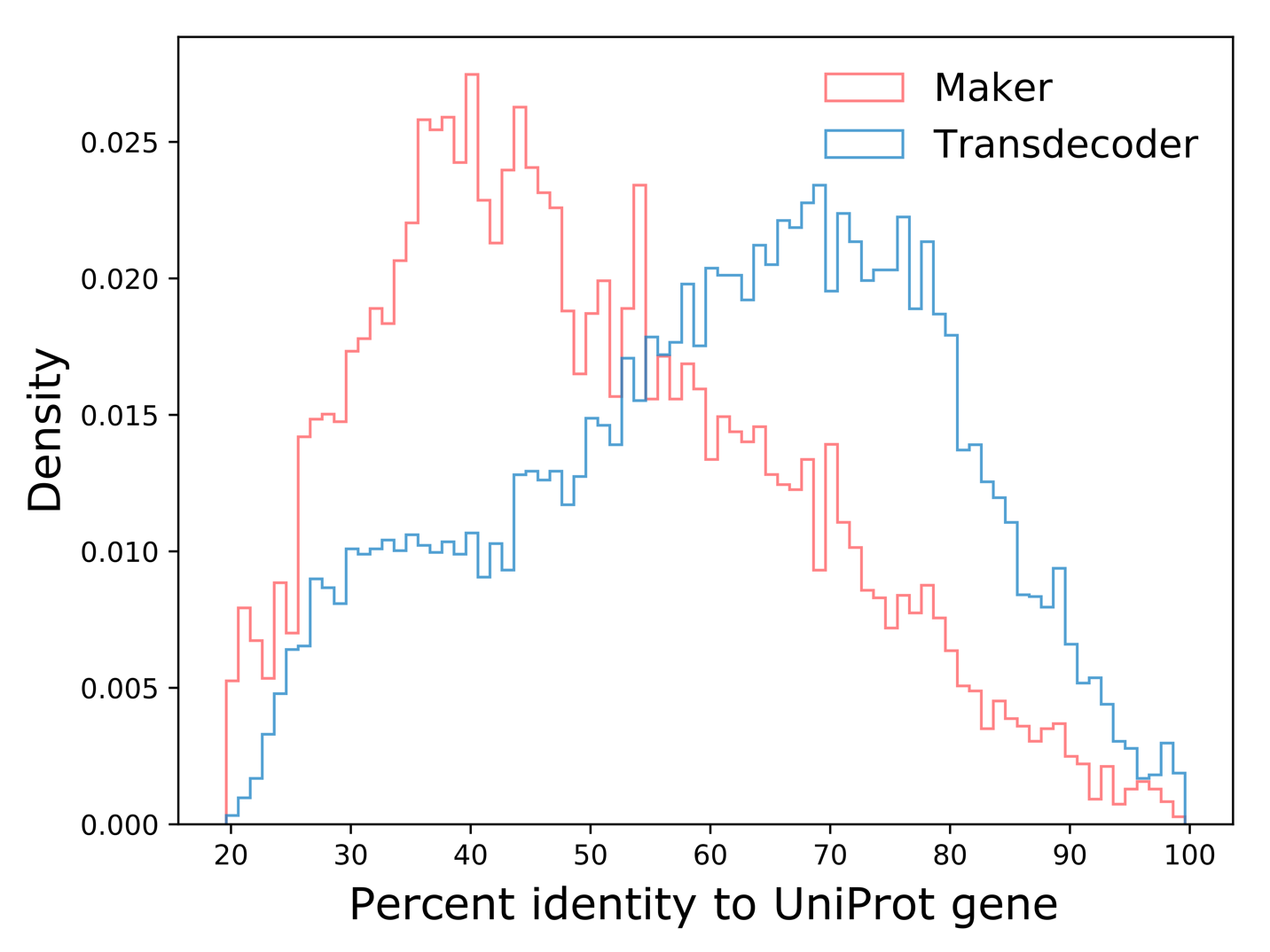


**Fig. S7. Comparison of inter-anchor distances and average Ks values in hop and hemp syntenic blocks. A)** Boxplot showing the percentage of syntenic blocks on a genome-wide scale that are composed of anchor genes, non-anchor genes, LTRs per syntenic block, or no annotated feature. **B)** This scatter plot shows the total inter-anchor distance for each syntenic block in hop syntenic blocks and hemp syntenic blocks, corresponding to the distance between anchor genes. **C)** This scatter plot shows the average Ks value per syntenic block on the x-axis and the number of genes in the corresponding syntenic block on the y-axis.


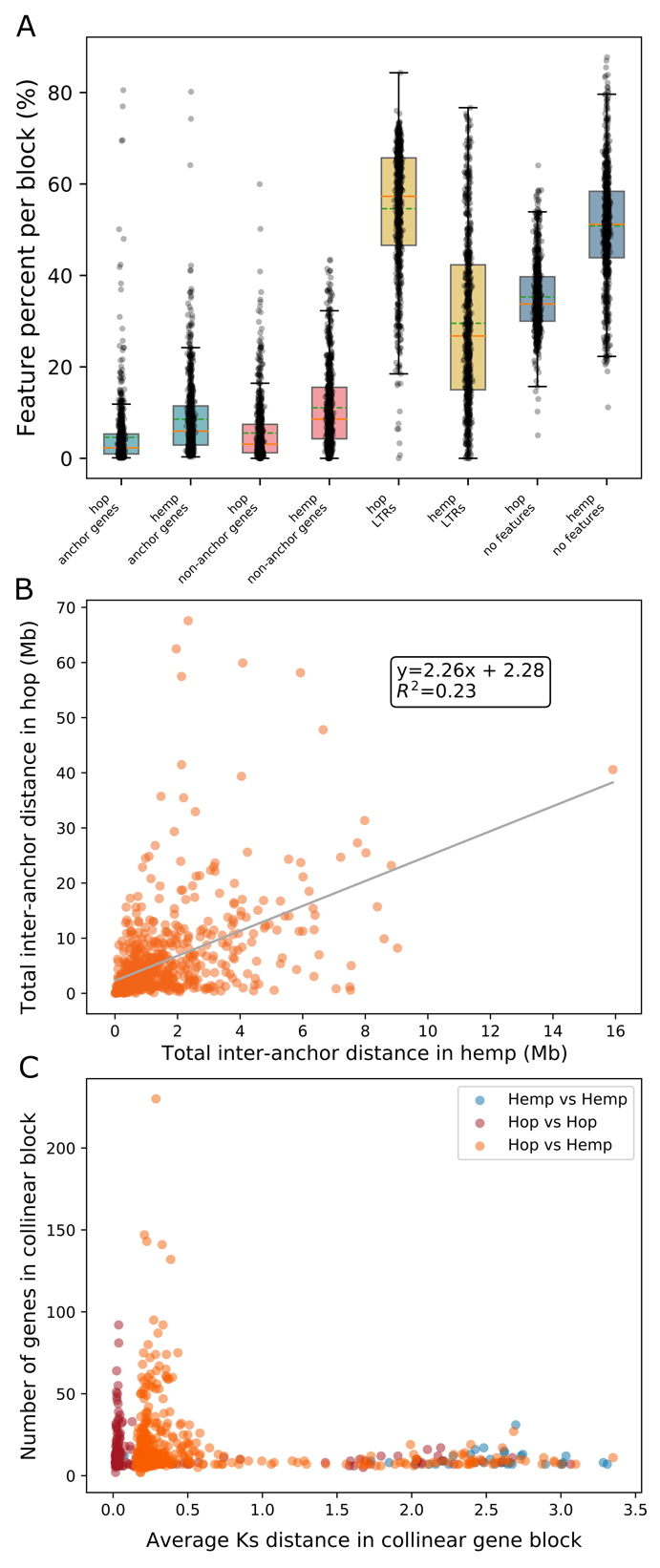


**Fig. S8. Functional enrichment of GO terms in hop vs hop syntenic blocks.** This scatter plot shows functional enrichment and depletion for hop genes in syntenic gene blocks present within the largest ten scaffolds in hop. The labeled GO terms are among the most statistically significant GO terms that have an observed count of at least six. The gradient for the color bar is shaded according to Q-value. Functional enrichment for **A)** biological processes; **B)** cellular components; and **C)** molecular function.


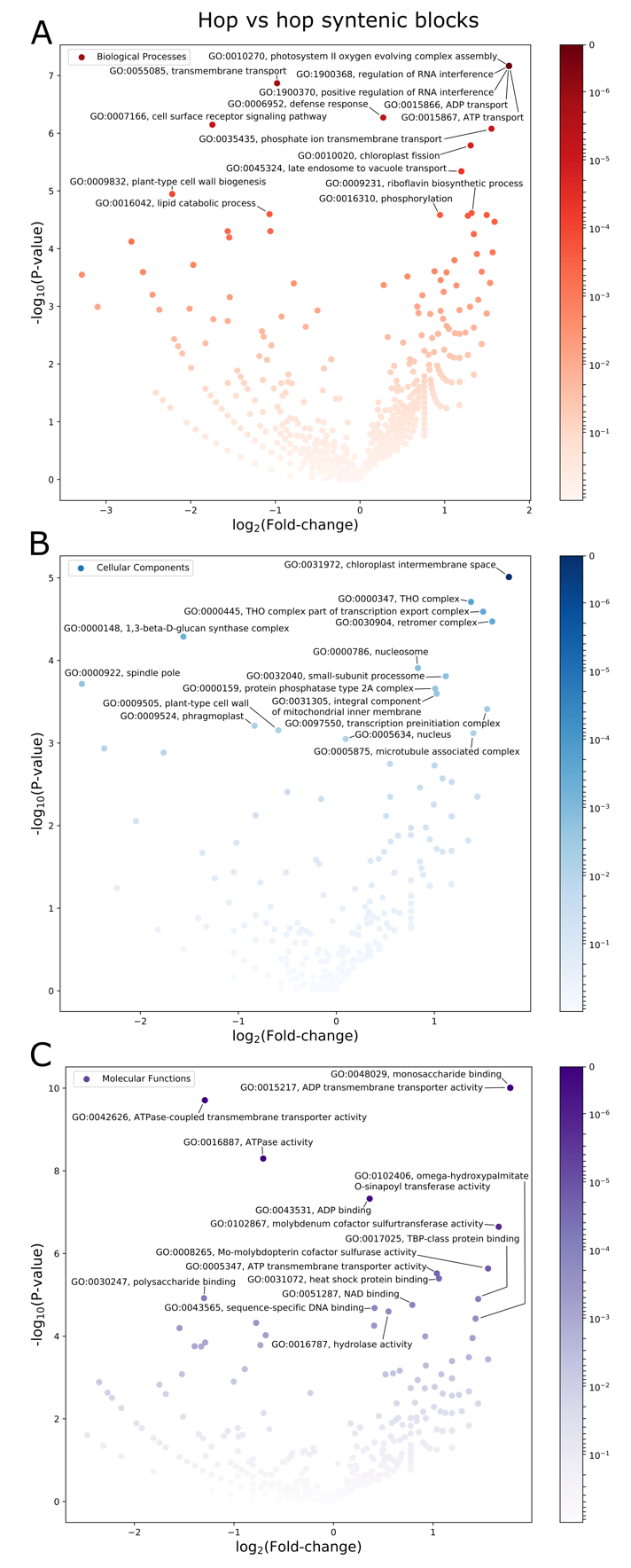


**Fig. S9.** **Functional enrichment of GO terms in hop vs hemp syntenic blocks.** This scatter plot shows functional enrichment and depletion for hop genes in syntenic gene blocks shared between the largest ten scaffolds in hop and largest ten scaffolds in hemp. The labeled GO terms are among the most statistically significant GO terms that have an observed count of at least six. The gradient for the color bar is shaded according to Q-value. Functional enrichment for **A)** biological processes; **B)** cellular components; and **C)** molecular function.

**
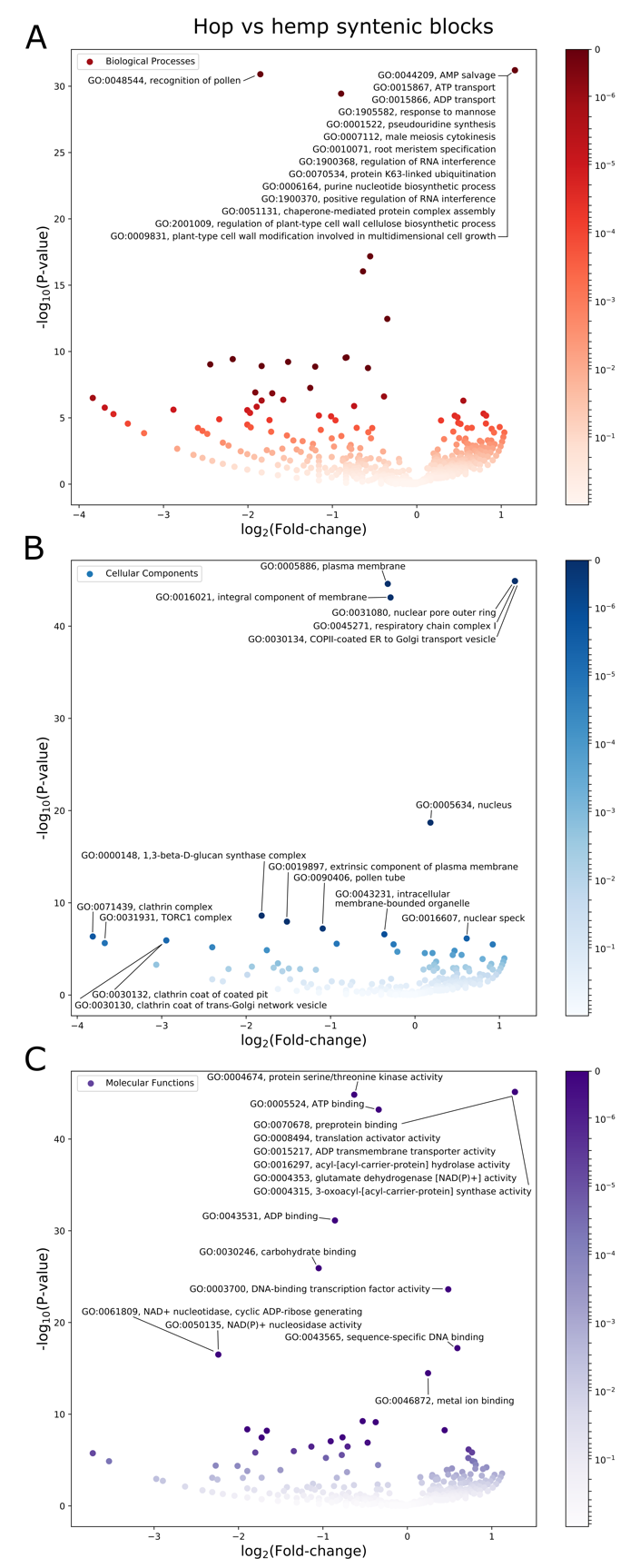
**

**Fig. S10. Total number of defense and terpene genes in syntenic blocks. A)** In hop vs hop syntenic blocks, chromosome 5 (Scaffold_172) has the most defense genes (175) along with five terpene genes; **B)** in hop vs hemp syntenic blocks, chromosome 8 (Scaffold_49) has the most defense genes (172) along with eight terpene genes.

**
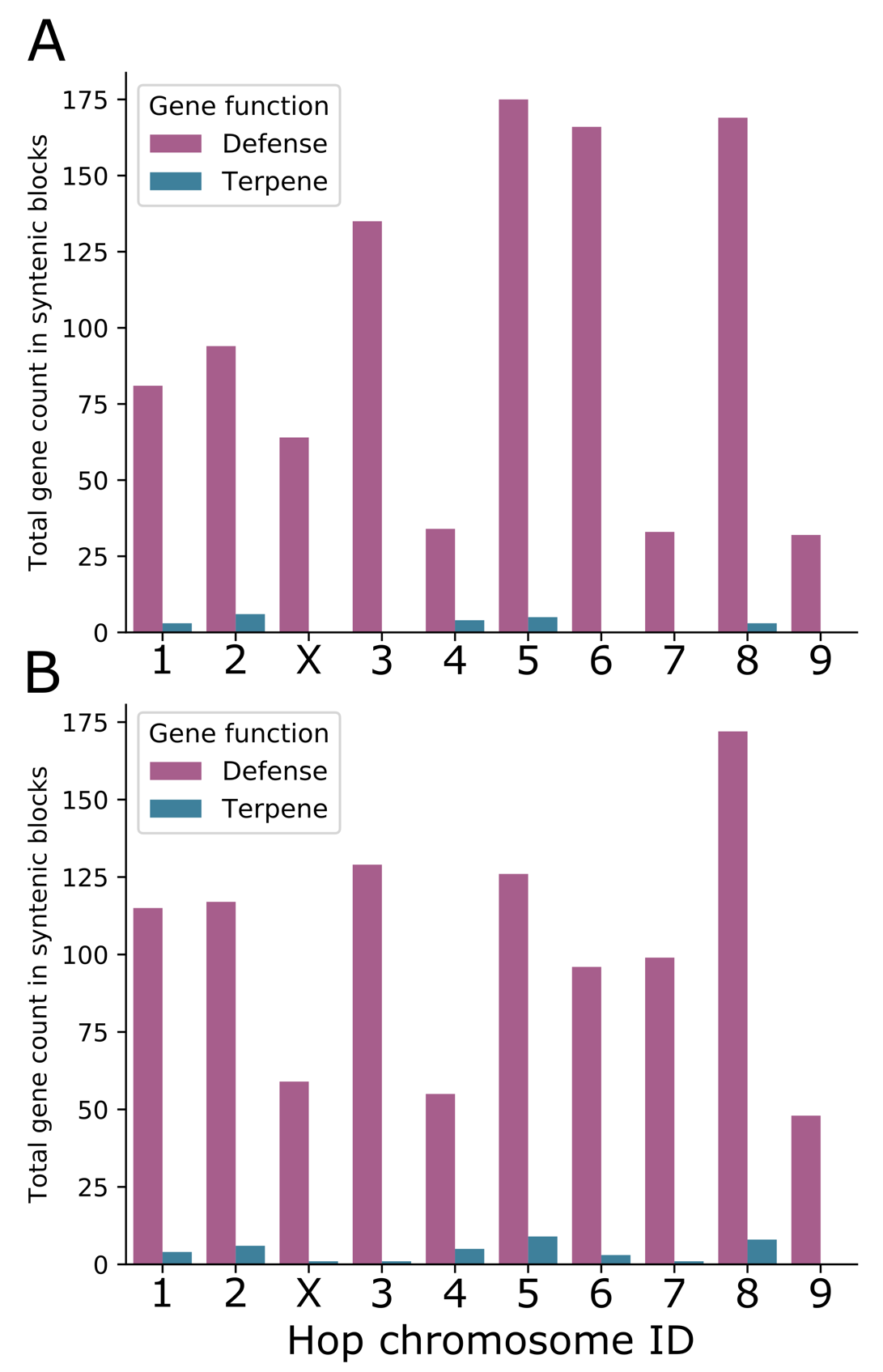
**

**Fig. S11. OrthoFinder results.** Bar charts showing the resulting statistics from OrthoFinder. **A)** Transdecoder gene models only; **B)** Transdecoder gene models and all MAKER gene models; and **C)** Transdecoder gene models and MAKER genes containing similarity to a known UniProt gene or Pfam domain. The three categories are shown for each of the eight species on the x-axis. The categories include the percentage of genes in all orthogroups, the percentage of orthogroups containing a given species, and the percentage of species-specific orthogroups. Our goal from this comparison was to find the set of gene models that maximized the number of orthogroups containing species, while minimizing species-specific orthogroups without similarity to a known gene.


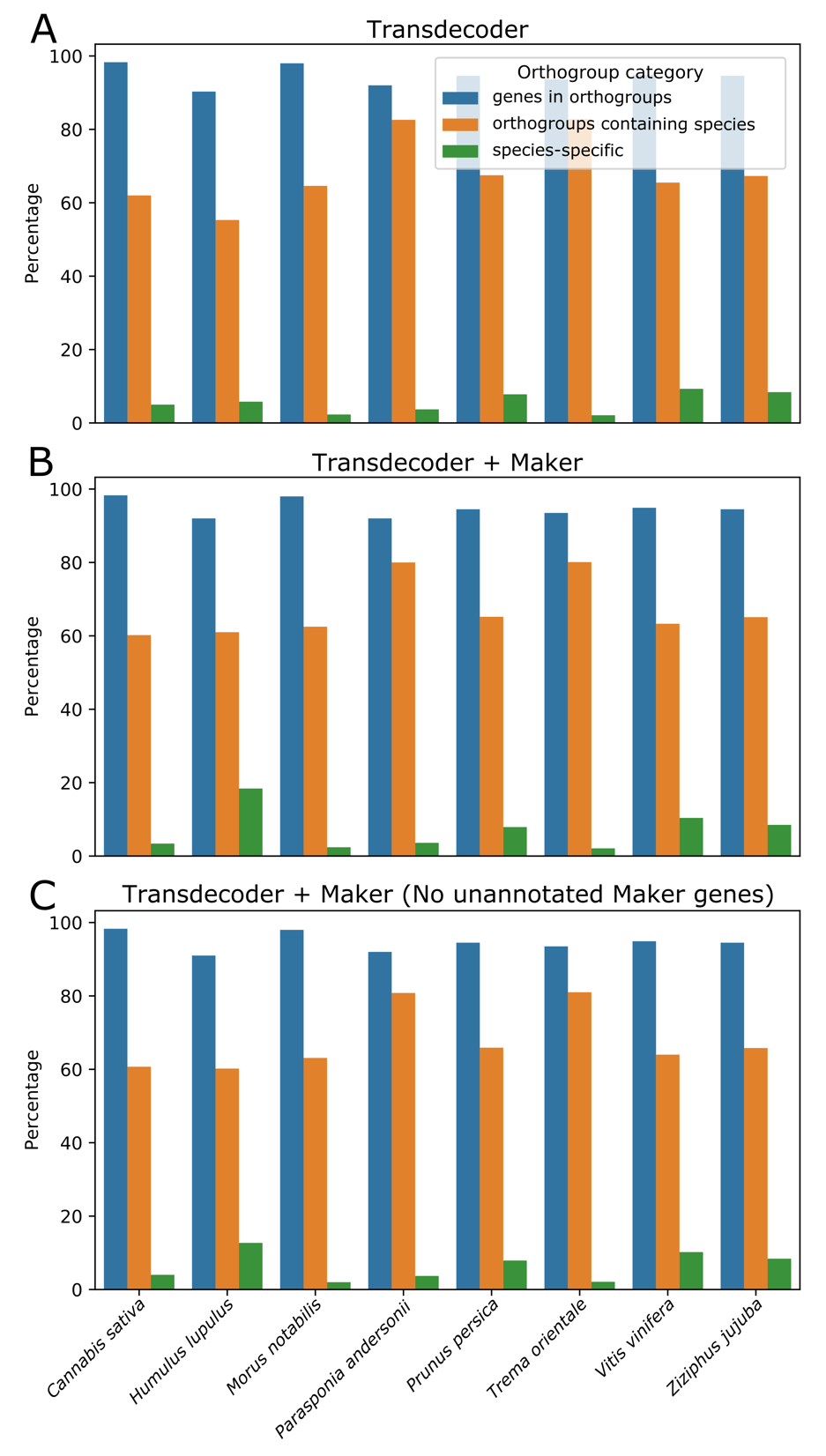


**Fig. S12. MCMCTree convergence. A)** Assessment of convergence by comparing the posterior means between two replicate runs for the strict molecular clock model (clock1). **B)** Assessment of convergence by comparing the posterior means between two replicate runs for the independent log-normally distributed relaxed-clock model (clock2). **C)** Trace of the posterior mean for the log-likelihood for the strict molecular clock. Translucent portion of trace denotes burn-in. The effective sample size (ESS) is 9,001. **D)** Trace of the posterior mean for the log-likelihood for the independent log-normally distributed relaxed-clock model. Translucent portion of trace denotes burn-in. The ESS is 8,885.


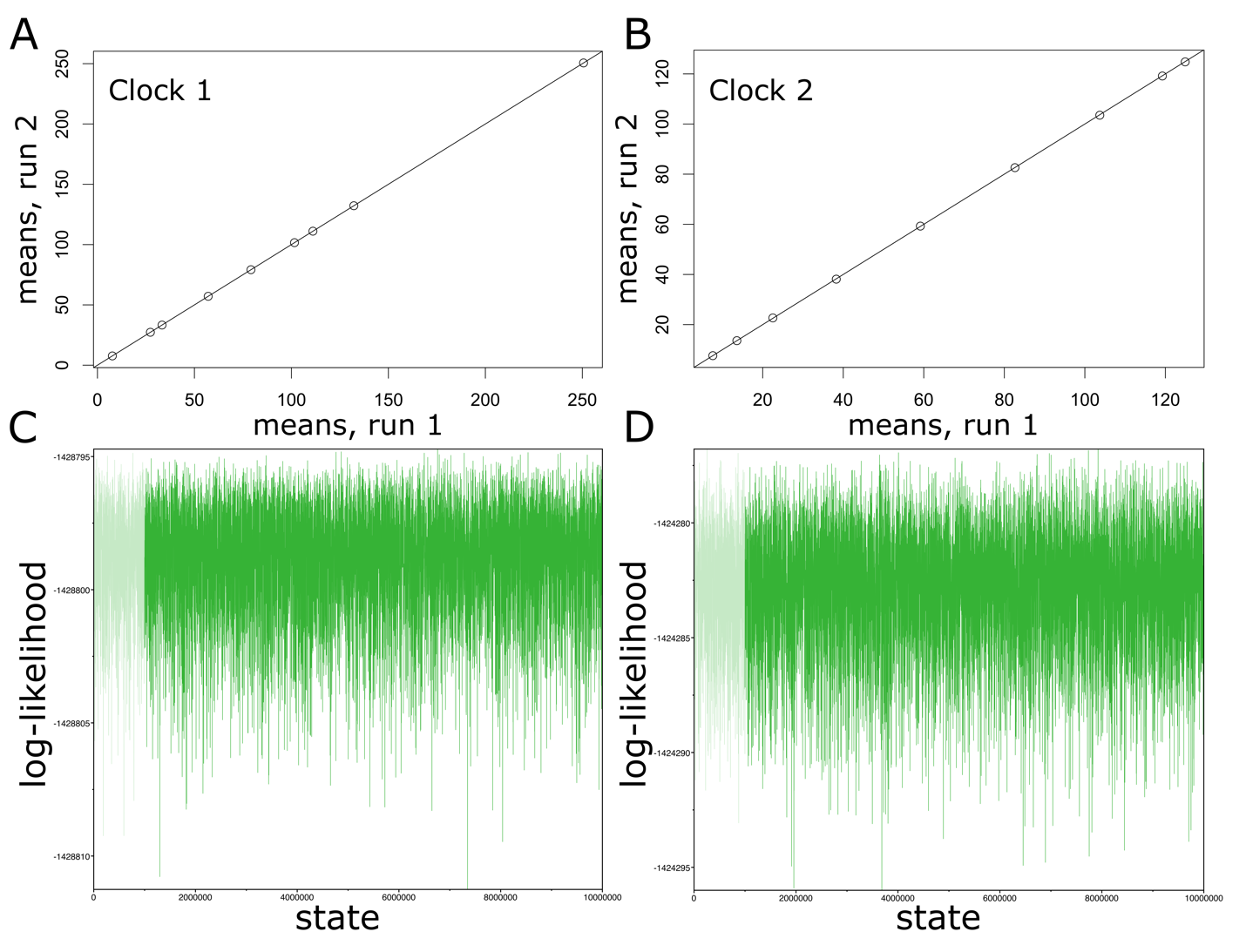


**Fig. S13. Estimation of time divergence using RAxML, r8s, and treePL.** The species trees for both time trees were generated with RAxML using 1,000 bootstrap replicates. **A)** r8s. **B)** treePL.


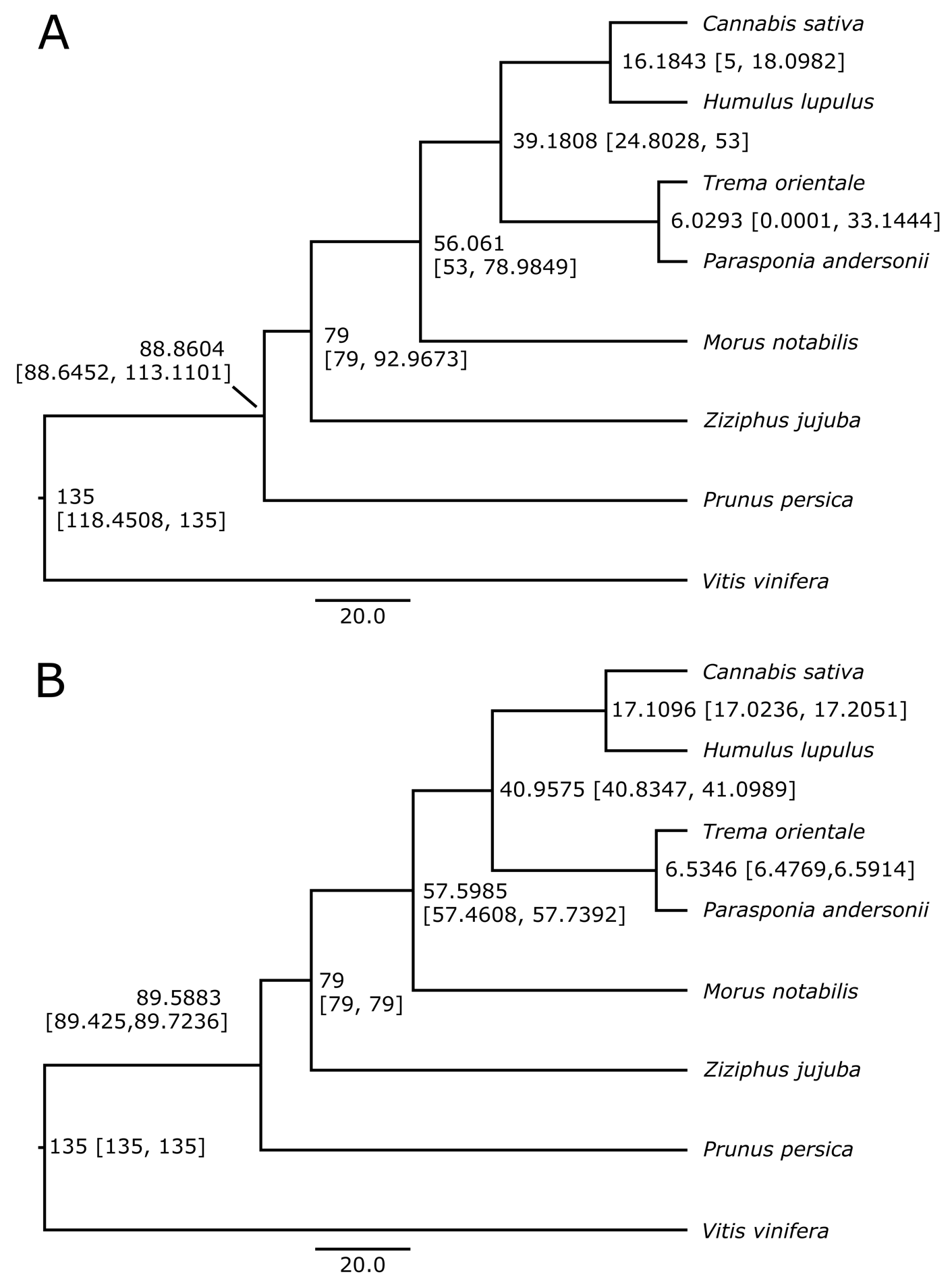


**Table S1.** **Statistics about Hi-C libraries and HiRise assembly**

| Number of breaks made to input assembly by HiRise | 1,027 |
| --- | --- |
| Number of joins made by HiRise | 8,131 |
| Library 1 stats | 156M read pairs; 2x150 bp |
| Library 2 stats | 92M read pairs; 2x150 bp |
| Library 3 stats | 143M read pairs; 2x150 bp |
| Library 4 stats | 67M read pairs; 2x150 bp |

**Table S2.** **Analysis of polished assembly quality**

| Substitution Errors | 961,874 bp |
| --- | --- |
| Insertion/Deletion Errors | 421,693 bp |
| Assembly Size | 3,712,781,139 bp |
| Consensus Quality | 99.9627% |

**Table S3. Comparative assembly statistics**

| Category | PacBio assembly | Dovetail Hi-C polished assembly (full assembly) |
| --- | --- | --- |
| Assembly size (bp) | 3,711,963,939 bp | 3,713,677,344 bp |
| Number of scaffolds | 8,661 | 1,533 |
| Number of scaffolds > 1kb | 8,661 | 1,533 |
| Largest scaffold (bp) | 8,249,941 bp | 476,374,791 bp |
| N50 (Mb) | 672,603 bp | 345,299,309 bp |
| N90 (Mb) | 221,394 bp | 185,200,997 bp |
| GC content (%) | 39.14% | 39.13% |
| Assembly gaps | 0 | 8172 |
| Percent of genome in gaps | 0 | 0.02% |

**Table S4. Repeat percentages relative to total repeat content and assembly length**

| Repeat type | Length (bp) | Percentage of repeat content | Percentage of assembly (3,713,677,344 bp) | Repeat annotation source |
| --- | --- | --- | --- | --- |
| LTR | 2,307,539,398 | 96.706 | 62.136 | *de novo* identification |
| DNA | 6,894,776 | 0.289 | 0.186 | MIPS PlantDB |
| LINE | 963,253 | 0.04 | 0.026 | MIPS PlantDB |
| SINE | 199 | 0.0 | 0.0 | MIPS PlantDB |
| Simple | 65,506,731 | 2.745 | 1.764 | MIPS PlantDB |
| Mobile element | 4,650,335 | 0.195 | 0.125 | MIPS PlantDB |
| rRNA | 204,089 | 0.009 | 0.005 | MIPS PlantDB |
| Other | 390,728 | 0.016 | 0.011 | MIPS PlantDB |
| Total repeat content | 2,386,149,509 | 100.0 | 64.253 | -- |

**Table S5. Assembly BUSCO results**

| Category | Full assembly or largest 10 scaffolds | Database | % Total complete | % Single-copy complete | % Duplicated complete | % Fragmented | % Missing |
| --- | --- | --- | --- | --- | --- | --- | --- |
| Polished, masked | full | Embryophyta (1,614) | 95.9 | 88.2 | 7.7 | 1.3 | 2.8 |
| **Polished, masked** | **10** | **Embryophyta (1,614)** | **96.0** | **92.6** | **3.4** | **1.4** | **2.6** |
| Polished, masked | full | Viridiplantae (425) | 97.0 | 90.4 | 6.6 | 0.7 | 2.3 |
| Polished, masked | 10 | Viridiplantae (425) | 97.5 | 94.4 | 3.1 | 0.7 | 1.8 |
| Polished, unmasked | full | Embryophyta (1,614) | 94.9 | 87.7 | 7.2 | 1.3 | 3.8 |
| Polished, unmasked | 10 | Embryophyta (1,614) | 94.3 | 91.3 | 3.0 | 1.4 | 4.3 |
| Unpolished, unmasked | full | Embryophyta (1,614) | 92.0 | 85.5 | 6.5 | 2.3 | 5.7 |

**Table S6.** **Estimated genome sizes of *Humulus* and closely related species**

| Species or variety | Estimated genome size (pg) | Estimated haploid genome size (Gb) | Reference |
| --- | --- | --- | --- |
| *Humulus lupulus* var. *cordifolia* | 2.9 (1C) | 2.842 | (Zonneveld *et al.*, 2005; Leitch *et al.*, 2019) |
| *H. lupulus* var. *lupulus* cv. Lubelski | 5.598±0.044 (2C) | 2.743 | (Grabowska-Joachimiak *et al.*, 2006) |
| *H. lupulus* var. *lupulus* cv. Shinshu Wase | 5.62 (2C) | 2.57 | (Natsume *et al.*, 2015) |
| *H. lupulus* cv. Brambling Cross | 3.1 (1C) | 2.989 | (Zonneveld *et al.*, 2005) |
| *H. lupulus* var. *neomexicanus* | 6.064±0.048 (2C) | 2.971 | (Grabowska-Joachimiak *et al.*, 2006) |
| *H. scandens (*synonymous with *H. japonicus)* | 3.208±0.028 (Grabowska-Joachimiak *et al.*, 2006)  (2C); 1.7 (Zonneveld *et al.*, 2005; Leitch *et al.*, 2019) (1C) | 1.572-1.666 | (Zonneveld *et al.*, 2005; Grabowska-Joachimiak *et al.*, 2006; Leitch *et al.*, 2019) |

**Table S7.** **Hop genome heterozygosity and repeat content based on short-read DNA sequencing**

| Results from GenomeScope 2.0 (ploidy=2; k-mer=21) | | |
| --- | --- | --- |
| Property | Min | Max |
| Homozygous (aa) | 94.5323% | 95.4073% |
| Heterozygous (ab) | 4.59269% | 5.4677% |
| Genome Haploid Length | 1,572,209,199 bp | 1,658,246,355 bp |
| Genome Repeat Length | 1,207,266,703 bp | 1,273,332,843 |
| Genome Unique Length | 364,942,496 bp | 384,913,512 |
| Model Fit | 33.166% | 92.0062% |
| Read Error Rate | 0.480394% | |

**Table S8.** **Linkage group statistics for the mapping population USDA 2017014**

| Linkage  Group | #Markers | Map Size | Average Gap Size | Biggest Gap Size | #Unique Positions |
| --- | --- | --- | --- | --- | --- |
|  |  | ---------------------cM---------------------- | | |  |
| 1 | 487 | 128.419 | 0.26 | 2.731 | 487 |
| 2 | 311 | 94.254 | 0.3 | 2.505 | 311 |
| 3 | 327 | 125.717 | 0.39 | 3.786 | 326 |
| 4 | 406 | 121.541 | 0.3 | 1.435 | 405 |
| 5 | 520 | 110.051 | 0.21 | 0.838 | 520 |
| 6 | 365 | 119.981 | 0.33 | 2.983 | 365 |
| 7 | 209 | 164.823 | 0.79 | 15.049 | 209 |
| 8 | 627 | 84.314 | 0.13 | 0.421 | 627 |
| 9 | 399 | 135.052 | 0.34 | 1.735 | 398 |
| 10 | 439 | 185.377 | 0.42 | 1.513 | 438 |
| All | 4,090 | 1,269.529 | 0.35 | 15.049 | 4086 |

**Table S9. Sex-associated genetic map for mapping population USDA 2017014 (provided as a separate file)**

**Table S10. Sex-associated linkage disequilibrium map for mapping population USDA 2017014 (provided as a separate file)**

**Table S11. Transdecoder gene model results**

| Transdecoder gene models | |
| --- | --- |
| **Category** | **Count** |
| Total gene models (complete + fragmented) | 57,684 |
| Complete protein-coding transcripts | 38,711 |
| Longest complete open reading frame (ORF) in full assembly | 21,698 |
| Longest complete open reading frame (ORF) in ten largest scaffolds | 20,581 |
| Lacking start codon | 11,691 |
| Lacking stop codon | 4,395 |
| Lacking start and stop codons | 2,887 |

**Table S12.** **Gene model BUSCO results**

| Category | Full assembly or largest 10 scaffolds | Database | % Total complete | % Single-copy complete | % Duplicated complete | % Fragmented | % Missing |
| --- | --- | --- | --- | --- | --- | --- | --- |
| Gene models (Transdecoder + MAKER) | full | Embryophyta (1614) | 78.3 | 57.7 | 20.6 | 6.9 | 14.8 |
| **Gene models (Transdecoder + MAKER)** | **10** | **Embryophyta (1614)** | **76.5** | **58.9** | **17.6** | **6.8** | **16.7** |
| Gene models (Transdecoder) | full | Embryophyta (1614) | 74.2 | 55.0 | 19.2 | 6.4 | 19.4 |
| Gene models (Transdecoder) | 10 | Embryophyta (1614) | 72.9 | 56.4 | 16.5 | 6.4 | 20.7 |

**Table S13. Gene statistics**

| **Category** | **Transdecoder + MAKER combined** | **Transdecoder** | **MAKER** |
| --- | --- | --- | --- |
| Number of protein-coding genes | 76,595 | 21,698 | 54,897 |
| Average length of protein (amino acids) | 390.96 | 392.74 | 360.74 |
| Total number of genes with similarity to UniProt Embryophyta gene or Pfam domain | 26,654 | 17,281 | 9,373 |
| Number of genes with similarity to UniProt Embryophyta | 23,583 | 15,445 | 8,138 |
| Number of genes with Pfam domain | 24,540 | 16,367 | 8,173 |
| Number of repeat-associated genes | 34,840 | 667 | 34,173 |
| Number of genes lacking similarity to a known UniProt gene or Pfam domain | 15,101 | 3,750 | 11,351 |

**Table S14. Number of genes with GO terms**

| **GO term category** | **Number of genes associated with GO term category** |
| --- | --- |
| Biological Processes | 19,147 |
| Cellular Components | 20,674 |
| Molecular Function | 19,385 |

**Table S15.** **Pfam repeat-associated domains** (this table is provided as a separate file)

**Table S16.** **Conditional repeat Pfam domains**

| Pfam Accession ID | Pfam Accession Description |
| --- | --- |
| PF04434.18 | SWIM zinc finger |
| PF16588.6 | C2H2 zinc-finger |
| PF02892.16 | BED zinc finger |
| PF13917.7 | Zinc knuckle |
| PF14372.7 | Domain of unknown function (DUF4413) |
| PF09331.12 | Domain of unknown function (DUF1985) |
| PF13962.7 | Domain of unknown function |
| PF00385.25 | Chromo (CHRromatin Organisation MOdifier) domain |
| PF02902.20 | Ulp1 protease family, C-terminal catalytic domain |
| PF18907.1 | Family of unknown function (DUF5662) |
| PF00574.24 | Clp protease |
| PF11604.9 | Copper binding periplasmic protein CusF |
| PF13634.7 | Nucleoporin FG repeat region |
| PF00230.21 | Major intrinsic protein |
| PF05193.22 | Peptidase M16 inactive domain |

**Table S17. MCSCanX collinearity output file (provided as a separate file)**

**Table S18. Syntenic blocks with defense and terpene-associated genes in ten largest scaffolds**

| Category | Hop vs hop | Hop vs hemp |
| --- | --- | --- |
| Total number of syntenic blocks | 264 | 725 |
| Total number of genes in syntenic blocks | 7,837 | 11,698 |
| Number of syntenic blocks with defense-associated genes | 188 | 382 |
| Number of defense-associated genes that occur in syntenic blocks | 983 | 1,016 |
| Expected number of syntenic blocks with terpene genes | (264*(11/264.0)*(188/264.0))=7.8 | (725*(32/725.0)*(382/725.0))= 16.9 |
| Observed number of syntenic blocks with terpene genes | 11 | 32 |
| Number of terpene genes that occur in syntenic blocks | 21 | 38 |
| Number of syntenic blocks with both defense and terpene-associated genes | 11 | 27 |
| Number of genes in syntenic blocks that are dually associated with defense and terpene function | 4 | 6 |

**Table S19. Genes with defense-associated GO terms in ten largest scaffolds (provided as a separate file)**

**Table S20. Genes with terpene-associated GO terms in ten largest scaffolds (provided as a separate file)**

**Methods S1.**

**SNP identification and filtration**

To identify sex-associated SNPs, we used the TASSEL 5.0 Pipeline (Glaubitz *et al.*, 2014) on default settings. The polished Cascade assembly was used as a reference genome for alignment with bowtie2 (Langmead & Salzberg, 2012). Approximately 1.3 million SNPs were identified and pre-filtered using default settings. The initial set of markers used for genetic mapping were filtered iteratively in TASSEL 5.52 (Bradbury *et al.*, 2007), alternating between incremental increases in stringency of genotypes or markers. Ultimately, a set of 55,214 markers across all samples (281 offspring and the two parental genotypes: female cultivar Comet and USDA male accession 19058M) were selected with 15% missing markers allowed, minor allele frequency of at least 0.2, and all selected genotypes having at least 80% presence across all samples. Missing marker data was then imputed using linkage disequilibrium (LD) KNNi imputation (Money *et al.*, 2015). The resulting data were then separated into separate pseudo-chromosomal groups and each group was exported as a VCF file.

LD map shows better concordance between physical and genetic positions than the genetic map. The LD map uses LD units to estimate LD between two physical markers and does not estimate the position of the marker. In contrast, the genetic map estimates the position based upon recombination, as well as genetic distance between markers using those same recombination values. Recombination estimates the number of offspring possessing a given genotype and then uses this information to calculate a value that has many assumptions. One assumption is absence of LD or reduced levels of LD. We can meet neither of these assumptions in hop due to heterozygosity, multivalent formation, and duplicated regions of the genome (Easterling *et al.,* 2018, Ono 1962, Zhang *et al.,* 2017). Because we cannot meet these assumptions, the LD map is a more appropriate measure of genetic mapping. In our case, Comet and 19058M are not related to Cascade and may potentially have rearrangements of the genome that could potentially influence the estimation of genetic position based on recombination.

**Haplotype-tagging SNPs (htSNPs) and SNPtag data**

SNPs from all 10 pseudo-chromosomes were split into individual sets with each set representing a single pseudo-chromosome. Pseudo-chromosome-specific data sets (in VCF format) were imported into JMP Genomics (JMP®, SAS Institute Inc., Cary, NC, 1989–2021., 1989-2021) and converted to numeric data. We then identified linkage disequilibrium blocks (using “LD Block Creation”) for initial bin-formation. Maximum range of LD Block creations was set to “20 markers,” the Haplotype Estimation Model was set to “EST,” and the Block options were set as follows: MAF = 0.1; all other values are default. We subsequently ran, “Haplotype Estimation” on the resulting data sets from LD Block Creation and used the resulting data sets for identification of haplotype-tagging SNPs (“htSNP analysis”). The two output files resulting from “htSNP” analyses consisted of one file containing all possible SNPs—both those representing bins as well as SNPs not located within a linkage block (singletons)—and a file with just SNPs representing LD bins. The latter file was then filtered to have just one representative SNP per LD bin (SNPtags). This filtered file was subsequently used for development of the genetic maps for each of the pseudo-chromosomes except for pseudo-chromosome 6. In this one case, the resulting data set exhibited fewer linkage blocks that were larger on average than the other pseudo-chromosomes. As a result, we added singletons to the starting file for development of a genetic map for this linkage group.

**Genome size and heterozygosity**

We used KmerGenie version 1.7051 to perform the k-mer distribution analysis, which determined an optimal k-mer size of 75 (Chikhi & Medvedev, 2014). We also estimated the heterozygosity of the genome using GenomeScope 2.0, which recommends a k-mer size of 21 for most genomes (Ranallo-Benavidez *et al.*, 2020). We used KMC (Kokot *et al.*, 2017) to count k-mers as input for GenomeScope 2.0. To perform these analyses, we used the short-read DNA sequencing from Cascade.

**RNA extraction and processing for RNA-seq**

RNA was extracted from gland and leaf hop tissues using a Qiagen RNeasy kit. The lysis buffer in the kit was replaced with a buffer of 4M guanidine isothiocyanate, 0.2 M sodium acetate pH 5.0, 25mM EDTA, 2.5% (w/v) PVP-40, and 1% (v/v) Beta-mercaptoethanol. The quality of the RNA was tested on an Agilent Bio-analyzer. The extracted total RNA passing QC was used with an Illumina TruSeq RNA library preparation kit. The resulting cDNA libraries were sequenced on an Illumina HiSeq at Illumina's California campus.

**Gene model development with Transdecoder**

To predict protein-coding transcripts, we used Transdecoder-v5.5.0 (Furuno *et al.*, 2003; Haas *et al.*, 2013). We aligned RNA-seq from lupulin glands, leaf, meristem, stem tissues, as well as hop cones during critical developmental stages (Padgitt-Cobb *et al.*, 2021; Eriksen *et al.*, 2021) to the Cascade Dovetail assembly, and then assembled transcripts. Alignment of RNA-seq to the assembly was performed with hisat2 version 2.2.0 (Kim *et al.*, 2019; Xu *et al.*, 2020), followed by transcript assembly with StringTie v1.3.3b (Pertea *et al.*, 2015; Xu *et al.*, 2020), and finally, merging of transcript-specific assemblies with the ‘stringtie --merge’ option.

Transdecoder identifies the coding DNA sequence (CDS) for each transcript from the longest open reading frame (ORF) (Furuno *et al.*, 2003; Xu *et al.*, 2010). Transdecoder makes its decision by incorporating the length of the ORF, a log-likelihood score, and a position-specific scoring matrix (PSSM) to refine the transcript boundaries. Transdecoder categorizes ORFs into four types, depending on the presence of start and stop codons, including “complete,” “5prime_partial,” “3prime_partial,” and “internal.” Transcripts identified as “5prime_partial” do not have a start codon, “3prime_partial” do not have a stop codon, and “internal” do not contain start or stop codons.

We extracted transcripts from the genome assembly with the following command: “TransDecoder-v5.5.0/util/gtf_genome_to_cdna_fasta.pl stringtie_merged.gtf cascadeAssembly.fasta > transcripts.fasta.” We generated a corresponding GFF file with the following command: “TransDecoder-v5.5.0/util/gtf_to_alignment_gff3.pl stringtie_merged.gtf > transcripts.gff3.”

Transdecoder proceeds in multiple steps. First, we extracted the longest ORF sequences by running the command, “TransDecoder.LongOrfs -S -t transcripts.fasta.” We then identified transcripts with similarity to UniProt Embryophyta protein sequences (38,747 sequences, accessed 08/24/2020) and Pfam domains (Pfam release 33.1). Next, we predicted genes with the command, “TransDecoder.Predict -t transcripts.fasta --retain_pfam_hits pfam.domtblout --retain_blastp_hits uniprot.blastp.” We generated a genome coordinate-centric GFF file with the following command:

“TransDecoder-v5.5.0/util/cdna_alignment_orf_to_genome_orf.pl transdecoder.gff3 transcripts.gff3 transcripts.fasta > transdecoder.genomeCentric.gff3.” For all subsequent analyses, we used the longest ORF per transcript. A minimum protein length of 100 amino acids was required, which is also the default length.

**Gene model development with MAKER**

The first step toward developing the set of gene models with MAKER involved generating alignment evidence. We used megablast 2.2.26 (Altschul *et al.*, 1990) to align ESTs from NCBI (25,692 sequences, accessed 11/12/2018) and TrichOME (Dai *et al.*, 2010) (22,959 sequences, accessed 03/28/2018) to the assembly. After obtaining initial alignments with megablast, we collected ESTs that aligned to the assembly, and performed a secondary alignment with est2genome (exonerate version 2.3.0) (Slater & Birney, 2005). We also generated protein alignments with *Cannabis sativa* (RefSeq; 33,639 sequences, accessed accessed 12/02/2020), *Prunus persica* (PLAZA (Van Bel *et al.*, 2018); 26,843 sequences, accessed 10/13/2020), *Ziziphus jujuba* (PLAZA (Van Bel *et al.*, 2018); 28,799 sequences, accessed 10/13/2020), and Embryophyta protein sequences (UniProt; 38,747 sequences, accessed 08/24/2020). For the protein sequences, we performed a first round of alignment with blastx 2.10.0+ and then performed a second round of alignment with exonerate protein2genome (exonerate version 2.3.0) (Slater & Birney, 2005) on the sequences that aligned to the assembly with blastx.

We also generated transcripts from RNA-seq for gene model prediction. Our approach began with alignment of RNA-seq to the assembly with hisat2 version 2.2.0 (Kim *et al.*, 2019; Xu *et al.*, 2020), followed by transcript assembly with StringTie v1.3.3b (Pertea *et al.*, 2015; Xu *et al.*, 2020), and finally, merging of transcript-specific assemblies from leaf, meristem, and stem tissues with cuffmerge 2011-03-17 (Trapnell *et al.*, 2010).

We generated final consensus gene models with MAKER (Cantarel *et al.*, 2008; Holt & Yandell, 2011; Campbell *et al.*, 2014; Xia *et al.*, 2019), using both Augustus-3.3.2 (Stanke *et al.*, 2006) and SNAP (Korf, 2004; Xu *et al.*, 2020) to perform gene model prediction. We trained Augustus by running BUSCO v4.1.1 in ‘long’ mode (Waterhouse *et al.*, 2018; Elbers *et al.*, 2019; Bohn *et al.*, 2021). Gene model development with MAKER proceeded in three steps. For the first round of MAKER, only the alignments were included as evidence in the config file (est2genome=1; protein2genome=1), to generate an initial set of models for training with SNAP. After the first round of MAKER, we trained SNAP. The resulting “snaphmm” file from SNAP was included in the second round of MAKER (est2genome=0; protein2genome=0). After the second round of MAKER, we trained SNAP again, producing an updated “snaphmm” file. The third round of MAKER included both the second-round “snaphmm” file from SNAP, as well as the Augustus models trained by BUSCO (De-la-Cruz *et al.*, 2021).

We used gffread to extract CDS and full transcripts (Pertea & Pertea, 2020). The detailed pipeline for gene prediction, including commands and MAKER config files, can be found at GeneModels/MakerGeneDevelopmentPipeline.md on the GitHub project page (<https://github.com/padgittl/CascadeDovetail>). A total set of 71,233 gene models was identified with MAKER.

**Combined gene models from Transdecoder and MAKER**

There were 5,196 MAKER gene models that overlapped with Transdecoder gene models, and the overlapping MAKER gene models were excluded in favor of Transdecoder gene models in subsequent analyses. After filtering for overlapping MAKER gene models and excluding proteins with fewer than 100 amino acids from the full assembly, we estimated 76,595 genes, including 21,698 genes from Transdecoder and 54,897 genes from MAKER. For the largest 10 scaffolds, the final set of gene models included 34,307 genes from MAKER and 20,581 genes from Transdecoder. Among the set of 76,595 genes, we identified 34,840 repeat-associated genes and 26,654 genes with similarity to a repeat- and non-repeat-associated gene (SI Table S13; Figure 2B).

**Density of genes and long terminal retrotransposons (LTRs)**

We calculated the density of genes and LTRs by counting the occurrence of gene and LTR sequences in a 5 Mb window.

**Gene model homology**

For evolutionary and orthology analyses, we obtained gene models from NCBI for *Cannabis sativa* (RefSeq assembly accession GCF_900626175.2), *Morus notabilis* (RefSeq assembly accession GCF_000414095.1), *Parasponia andersonii* (GenBank assembly accession GCA_002914805.1), *Trema orientale* (GenBank assembly accession GCA_002914845.1), and *Vitis vinifera* (RefSeq assembly accession GCF_000003745.3). We first removed genes annotated as pseudogenes or low quality and then included only the longest transcript per gene. We obtained the “selected transcripts” for *Prunus persica* and *Ziziphus jujuba* from PLAZA (accessed 10/13/2020), corresponding to the longest transcript. For defense and disease response-associated genes, we collected GO terms (Ashburner *et al.*, 2000) including keywords 'defense' or 'disease,' and then retrieved all UniProt genes associated with the selected GO terms.

**Repeat-associated gene models**

We followed an adapted version of our previously described pipeline to identify repeat-associated genes using both Pfam domains (Finn *et al.*, 2014) and UniProt genes. First, we collected 164 transposable element (TE)- and virus-associated Pfam domains (Pfam release 33.1) based on keyword search and literature (Supplemental Table 9). From UniProt, we downloaded sets of genes from bacteria (accessed 04/15/2021 using search term: taxonomy:"Bacteria [2]" AND reviewed:yes), viruses (accessed 04/15/2021 using search term: taxonomy:"Viruses [10239]" AND reviewed:yes), and TEs (accessed 04/15/2021 using search term: keyword:"Transposable element [KW-0814]" AND reviewed:yes). We also collected 12 transposon-associated genes from the set of 38,747 UniProt Embryophyta genes (accessed 08/24/2020 using search term: taxonomy:"Embryophyta [3193]" AND reviewed:yes).

We aligned the gene models to each set of UniProt genes in both directions using blastp (version 2.12.0+), applying an E-value threshold of less than 1e-3. We aligned the gene models to the Pfam domains with hmmscan (HMMER 3.3 (Nov 2019)), applying an E-value threshold of less than 1e-3.

We identified three categories of genes with similarity to Pfam domains: similarity only to non-repeat-associated domains, similarity only to repeat-associated domains, and similarity to both repeat- and non-repeat-associated domains. We further split the set of genes with similarity to both repeat- and non-repeat-associated domains into two categories. For the first category, we identified genes with domains that frequently occurred with repeat domains but did not have apparent repeat similarity when they occurred alone. We designated this set of domains as “conditional repeats” (Supplemental Table 10). For the remaining examples with both repeat- and non-repeat Pfam domains, without “conditional repeat” domains, we applied a coverage filter. If more than 30% of the length of a gene shared similarity with repeat domains, that gene was reassigned as a repeat gene (Ye & Zhong, 2015; Yang *et al.*, 2016).

To assign similarity to UniProt genes, we required a minimum percent identity based on alignment length, known as the “Twilight zone” of protein alignment (Rost, 1999), which provides a metric to quantify similarity between sequences with confidence. For Embryophyta UniProt genes, we also imposed a minimum query coverage of 20%, and for bacteria, virus, and TE UniProt genes, we imposed a minimum query coverage of 30%.

The final step in the filtering pipeline involved compiling all the sources of alignment evidence to assign genes to one of three categories: non-repeat, repeat, or unannotated. First, to assign a gene as non-repeat, we checked if genes with similarity to UniProt Embryophyta genes also shared similarity with any Pfam repeat domains. If all domain similarity was repeat-associated, the gene was assigned to the repeat category.

For genes with similarity to a UniProt TE gene, we checked first if these genes had similarity to a UniProt Embryophyta gene. If so, we required the gene with similarity to a TE to have greater query coverage to the TE than to the Embryophyta gene, to be reassigned as repeat.

For genes with similarity to UniProt bacteria and virus genes, we required that the gene did not already have similarity to an Embryophyta gene, to avoid removing genes with conserved function. Finally, we collected genes lacking any similarity to a UniProt gene or Pfam domain. We applied this pipeline to the set of hop gene models, as well as the other seven species in our orthology analysis. The detailed pipeline for gene annotation can be found at GeneModels/GeneAnnotationPipeline.md on the GitHub project page (https://github.com/padgittl/CascadeDovetail).

**Fossil calibration dates**

We used a fossil calibration date of 5-28 mya based on previously described calibration intervals from literature (Tiffney, 1986; Collinson, 1989; Zerega *et al.*, 2005; McPartland, 2018; Jin *et al.*, 2020). Most recently, Jin *et al.* restricted the *Humulus* constrained to 23 mya based on fruit fossil evidence of *Humulago reticulata* (Dorofeev) Doweld from Antropovo, Russia during the Oligocene (Collinson, 1989; Doweld, 2016), resulting in an estimated divergence date of 25.4 mya for *Humulus* and *Cannabis* (Jin *et al.*, 2020). Zerega *et al.* also set the fossil calibration date to 5-23 mya for *Humulus*, placing the divergence of *Humulus* and *Cannabis* at 21 mya (Zerega *et al.*, 2005). McPartland (McPartland, 2018) used a calibration date of 16-28 mya, placing *Humulus* and *Cannabis* divergence at the boundary of the early and late Oligocene (28 mya) based on morphological evidence for a "generalized" dispersal mechanism in *Humulus* and *Cannabis* (Tiffney, 1986). Calibration dates for *Trema* and *Parasponia* based on fossil evidence are not available to our knowledge.

We applied calibration dates for species outside of the Cannabaceae from TimeTree.org (Kumar *et al.*, 2017), while also verifying these dates with literature. He *et al.* estimated the divergence date of 63.5 mya for mulberry (*Morus notabilis*) and *Cannabis sativa* (He *et al.*, 2013). Early fossil evidence for the *Moraceae* family dates from the Eocene (56-33.9 mya). Based on molecular dating, the Rosidae clade has an estimated origination date between 123–93 mya (Magallón *et al.*, 2015; Sun *et al.*, 2016), and the estimated date for the divergence of *Vitis* is 115 mya (Fawcett *et al.*, 2009). The emergence of the eudicots is estimated to have occurred around 125 mya and is considered to be a reliable calibration date for molecular dating analyses (Doyle *et al.*, 1977; Hickey & Doyle, 1977; Doyle & Hotton, 1991; Fawcett *et al.*, 2009; Xiang *et al.*, 2017).

**MCMCTree parameters**

We used a likelihood-ratio test (LRT) to compare molecular clock models (Yang *et al.*, 2000) with the equation LRT=-2(ln(Ls)-ln(Lg)). L is the likelihood value, ln(x) is the natural logarithm, and s corresponds to the simpler model with fewer parameters than the general model g. We used the HKY85 substitution model (model=4) (Hasegawa *et al.*, 1985) and set the root age to <1.25, corresponding to 125 mya. Other parameters for MCMCTree include burnin = 50,000, sampfreq = 1,000, and nsample = 10,000. We evaluated the MCMC results with Tracer v1.7.2 (Fig. S11) (Suchard *et al.*, 2018; Dos Reis & Yang, 2019). The control file for MCMCTree can be viewed at the GitHub page under ‘TimeDivergenceEstimation’.

**Development of time trees with RAxML, r8s, and treePL**

To generate a species tree, we used RAxML version 8.2.12 (raxmlHPC -k -f a -m GTRGAMMA -p 12345 -x 12345 -q partition.txt -s allSpecies.fullCodon.phy -N 1000 -n fullCodon -o vitis_vinifera) (Stamatakis 2014). We required 1,000 bootstrap replicates (-N 1000) and labeling of branch lengths on each of the bootstrap trees (-k). The file called partition.txt was generated with concatenateFullCodons.py and can be found at the GitHub page under ‘TimeDivergenceEstimation’. The partitions.txt file contains information about the first and second codon positions, as well as the third codon position, which allows different models of substitution based on the codon position. To create allSpecies.fullCodon.phy we used Geneious Prime 2022.1.1 (https://www.geneious.com) to convert from our original fasta alignment file to relaxed-Phylip. RAxML produced two trees that we used to guide our analysis: RAxML_bestTree.fullCodon and RAxML_bootstrap.fullCodon. We performed the analysis with r8s and treePL on the best tree and bootstrap trees from RAxML.

To run r8s version 1.80 (Oct 15 2015) (Sanderson 2003) we accessed the r8s environment on the terminal by typing ‘r8s’ and then using the command, ‘execute best_tree.nex’ or ‘execute bootstrap_trees.nex’. The nexus files for this analysis can be found at the GitHub page under ‘TimeDivergenceEstimation’. To run treePL version 1.0 (Smith *et al.,* 2012, Maurin 2020) we used the command, ‘treePL config_best_tree.txt’ and ‘treePL config_bootstrap.txt’. The config files for treePL can be accessed at the GitHub page under ‘TimeDivergenceEstimation’.

The dated bootstrap trees from rs8 and treePL were summarized into consensus trees with TreeAnnotator v2.6.4, 2002-2021 (Bouckaert *et al.,* 2019). Parameters for TreeAnnotator included burnin percentage and posterior probability limit set to 0.0. We also selected the maximum clade credibility tree and mean node heights (Maurin 2020). The trees were visualized with FigTree v1.4.4 (Rambaut 2012).

**Supplemental References**

**Altschul SF, Gish W, Miller W, Myers EW, Lipman DJ**. **1990**. Basic local alignment search tool. *Journal of molecular biology* **215**: 403–410.

**Ashburner M, Ball CA, Blake JA, Botstein D, Butler H, Cherry JM, Davis AP, Dolinski K, Dwight SS, Eppig JT, *et al.*** **2000**. Gene Ontology: tool for the unification of biology. *Nature genetics* **25**: 25–29.

**Bohn J, Halabian R, Schrader L, Shabardina V, Steffen R, Suzuki Y, Ernst UR, Gadau J, Makałowski W**. **2021**. Genome assembly and annotation of the California harvester ant Pogonomyrmex californicus. *G3*  **11**.

**Bouckaert R, Vaughan TG, Barido-Sottani J, Duchêne S, Fourment M, Gavryushkina A, Heled J, Jones G, Kühnert D, De Maio N, Matschiner M.** BEAST 2.5: An advanced software platform for Bayesian evolutionary analysis. *PLoS computational biology*. 2019 Apr 8;15(4):e1006650.

**Bradbury PJ, Zhang Z, Kroon DE, Casstevens TM, Ramdoss Y, Buckler ES**. **2007**. TASSEL: software for association mapping of complex traits in diverse samples. *Bioinformatics*  **23**: 2633–2635.

**Campbell MS, Holt C, Moore B, Yandell M**. **2014**. Genome annotation and curation using MAKER and MAKER-P. *Current protocols in bioinformatics / editoral board, Andreas D. Baxevanis ... [et al.]* **48**: 4.11.1–39.

**Cantarel BL, Korf I, Robb SMC, Parra G, Ross E, Moore B, Holt C, Sánchez Alvarado A, Yandell M**. **2008**. MAKER: an easy-to-use annotation pipeline designed for emerging model organism genomes. *Genome research* **18**: 188–196.

**Chikhi R, Medvedev P**. **2014**. Informed and automated k-mer size selection for genome assembly. *Bioinformatics*  **30**: 31–37.

**Collinson ME**. **1989**. The fossil history of the Moraceae, Urticaceae (including Cecropiaceae), and Cannabaceae. *Evolution, systematics, and fossil history of the Hamamelidae* **2**: 319–339.

**Dai X, Wang G, Yang DS, Tang Y, Broun P, Marks MD, Sumner LW, Dixon RA, Zhao PX**. **2010**. TrichOME: a comparative omics database for plant trichomes. *Plant physiology* **152**: 44–54.

**De-la-Cruz IM, Hallab A, Olivares-Pinto U, Tapia-López R, Velázquez-Márquez S, Piñero D, Oyama K, Usadel B, Núñez-Farfán J**. **2021**. Genomic signatures of the evolution of defence against its natural enemies in the poisonous and medicinal plant Datura stramonium (Solanaceae). *Scientific reports* **11**: 882.

**Dos Reis M, Yang Z**. **2019**. Bayesian Molecular Clock Dating Using Genome-Scale Datasets. *Methods in molecular biology*  **1910**: 309–330.

**Doweld AB**. **2016**. Humulago, a Replacement Name for Fossil Humularia Dorofeev (Cannabaceae) non Extant Humularia Duvigneaud (Fabaceae). *Annales Botanici Fennici* **53**: 403–404.

**Doyle JA, Hotton CL**. **1991**. Diversification of early angiosperm pollen in a cladistic context. *Pollen and spores: patterns of diversification* **169**: 195.

**Doyle JA, Ja D, Others**. **1977**. Angiosperm pollen from the pre-Albian Lower Cretaceous of equatorial Africa.

**Easterling KA, Pitra NJ, Jones RJ, Lopes LG, Aquino JR, Zhang D, Matthews PD, Bass HW.** 3D molecular cytology of hop (Humulus lupulus) meiotic chromosomes reveals non-disomic pairing and segregation, aneuploidy, and genomic structural variation. *Frontiers in Plant Science*. 2018 Nov 1;9:1501.

**Elbers JP, Rogers MF, Perelman PL, Proskuryakova AA, Serdyukova NA, Johnson WE, Horin P, Corander J, Murphy D, Burger PA**. **2019**. Improving Illumina assemblies with Hi-C and long reads: An example with the North African dromedary. *Molecular ecology resources* **19**: 1015–1026.

**Eriksen RL, Padgitt-Cobb LK, Randazzo AM, Hendrix DA, Henning JA**. **2021**. Gene Expression of Agronomically Important Secondary Metabolites in cv. ‘USDA Cascade’ Hop (Humulus lupulus L.) Cones during Critical Developmental Stages. *Journal of the American Society of Brewing Chemists. American Society of Brewing Chemists*: 1–14.

**Fawcett JA, Maere S, Van de Peer Y**. **2009**. Plants with double genomes might have had a better chance to survive the Cretaceous–Tertiary extinction event. *Proceedings of the National Academy of Sciences of the United States of America* **106**: 5737–5742.

**Finn RD, Bateman A, Clements J, Coggill P, Eberhardt RY, Eddy SR, Heger A, Hetherington K, Holm L, Mistry J, *et al.*** **2014**. Pfam: the protein families database. *Nucleic acids research* **42**: D222–30.

**Furuno M, Kasukawa T, Saito R, Adachi J, Suzuki H, Baldarelli R, Hayashizaki Y, Okazaki Y**. **2003**. CDS annotation in full-length cDNA sequence. *Genome research* **13**: 1478–1487.

**Glaubitz JC, Casstevens TM, Lu F, Harriman J, Elshire RJ, Sun Q, Buckler ES**. **2014**. TASSEL-GBS: a high capacity genotyping by sequencing analysis pipeline. *PloS one* **9**: e90346.

**Gojobori T**. **1983**. Codon substitution in evolution and the ‘saturation’ of synonymous changes. *Genetics* **105**: 1011–1027.

**Grabowska-Joachimiak A, Sliwinska E, Pigula M, Skomra U, Joachimiak AJ**. **2006**. Genome size in Humulus lupulus L. and H. japonicus Siebold and Zucc.[Cannabaceae]. *Acta Societatis Botanicorum Poloniae* **75**: 207–214.

**Haas BJ, Papanicolaou A, Yassour M, Grabherr M, Blood PD, Bowden J, Couger MB, Eccles D, Li B, Lieber M, *et al.*** **2013**. De novo transcript sequence reconstruction from RNA-seq using the Trinity platform for reference generation and analysis. *Nature protocols* **8**: 1494–1512.

**Hasegawa M, Kishino H, Yano T**. **1985**. Dating of the human-ape splitting by a molecular clock of mitochondrial DNA. *Journal of molecular evolution* **22**: 160–174.

**Hellsten U, Khokha MK, Grammer TC, Harland RM, Richardson P, Rokhsar DS**. **2007**. Accelerated gene evolution and subfunctionalization in the pseudotetraploid frog Xenopus laevis. *BMC biology* **5**: 31.

**He N, Zhang C, Qi X, Zhao S, Tao Y, Yang G, Lee T-H, Wang X, Cai Q, Li D, *et al.*** **2013**. Draft genome sequence of the mulberry tree Morus notabilis. *Nature communications* **4**: 2445.

**Hickey LJ, Doyle JA**. **1977**. Early cretaceous fossil evidence for angiosperm evolution. *The Botanical review; interpreting botanical progress* **43**: 3–104.

**Holt C, Yandell M**. **2011**. MAKER2: an annotation pipeline and genome-database management tool for second-generation genome projects. *BMC bioinformatics* **12**: 491.

**Jin J-J, Yang M-Q, Fritsch PW, Velzen R, Li D-Z, Yi T-S**. **2020**. Born migrators: Historical biogeography of the cosmopolitan family Cannabaceae. *Journal of systematics and evolution* **58**: 461–473.

**JMP®, SAS Institute Inc. , Cary, NC, 1989–2021.** **1989-2021**. *JMP Genomics*.

**Kim D, Paggi JM, Park C, Bennett C, Salzberg SL**. **2019**. Graph-based genome alignment and genotyping with HISAT2 and HISAT-genotype. *Nature biotechnology* **37**: 907–915.

**Kokot M, Dlugosz M, Deorowicz S**. **2017**. KMC 3: counting and manipulating k-mer statistics. *Bioinformatics*  **33**: 2759–2761.

**Korf I**. **2004**. Gene finding in novel genomes. *BMC bioinformatics* **5**: 59.

**Kumar S, Stecher G, Suleski M, Hedges SB**. **2017**. TimeTree: A Resource for Timelines, Timetrees, and Divergence Times. *Molecular biology and evolution* **34**: 1812–1819.

**Langmead B, Salzberg SL**. **2012**. Fast gapped-read alignment with Bowtie 2. *Nature methods* **9**: 357–359.

**Leitch IJ, Johnston E, Pellicer J, Hidalgo O, Bennett MD**. **2019**. Angiosperm DNA C-values database (release 9.0, Apr 2019).

**Magallón S, Gómez-Acevedo S, Sánchez-Reyes LL, Hernández-Hernández T**. **2015**. A metacalibrated time-tree documents the early rise of flowering plant phylogenetic diversity. *The New phytologist* **207**: 437–453.

**Maurin KJ**. An empirical guide for producing a dated phylogeny with treePL in a maximum likelihood framework. *arXiv preprint* arXiv:2008.07054. 2020 Aug 17.

**McPartland JM**. **2018**. Cannabis Systematics at the Levels of Family, Genus, and Species. *Cannabis and cannabinoid research* **3**: 203–212.

**Money D, Gardner K, Migicovsky Z, Schwaninger H, Zhong G-Y, Myles S**. **2015**. LinkImpute: Fast and Accurate Genotype Imputation for Nonmodel Organisms. *G3*  **5**: 2383–2390.

**Natsume S, Takagi H, Shiraishi A, Murata J, Toyonaga H, Patzak J, Takagi M, Yaegashi H, Uemura A, Mitsuoka C, *et al.*** **2015**. The Draft Genome of Hop (Humulus lupulus), an Essence for Brewing. *Plant & cell physiology* **56**: 428–441.

**Ono T.** The wild hop native to Japan; ecology and morphology. *Bulletin of brewing science*. 1962(8).

**Padgitt-Cobb LK, Kingan SB, Wells J, Elser J, Kronmiller B, Moore D, Concepcion G, Peluso P, Rank D, Jaiswal P, *et al.*** **2021**. A draft phased assembly of the diploid Cascade hop (Humulus lupulus) genome. *The plant genome* **14**: e20072.

**Pertea G, Pertea M**. **2020**. GFF Utilities: GffRead and GffCompare. *F1000Research* **9**.

**Pertea M, Pertea GM, Antonescu CM, Chang T-C, Mendell JT, Salzberg SL**. **2015**. StringTie enables improved reconstruction of a transcriptome from RNA-seq reads. *Nature biotechnology* **33**: 290–295.

**Potato Genome Sequencing Consortium, Xu X, Pan S, Cheng S, Zhang B, Mu D, Ni P, Zhang G, Yang S, Li R, *et al.*** **2011**. Genome sequence and analysis of the tuber crop potato. *Nature* **475**: 189–195.

**Rambaut A.** FigTree v1. 4.

**Ranallo-Benavidez TR, Jaron KS, Schatz MC**. **2020**. GenomeScope 2.0 and Smudgeplot for reference-free profiling of polyploid genomes. *Nature communications* **11**: 1432.

**Ranwez V, Douzery EJP, Cambon C, Chantret N, Delsuc F**. **2018**. MACSE v2: Toolkit for the Alignment of Coding Sequences Accounting for Frameshifts and Stop Codons. *Molecular biology and evolution* **35**: 2582–2584.

**Rost B**. **1999**. Twilight zone of protein sequence alignments. *Protein engineering* **12**: 85–94.

**Rozenfeld C, Blanca J, Gallego V, García-Carpintero V, Herranz-Jusdado JG, Pérez L, Asturiano JF, Cañizares J, Peñaranda DS**. **2019**. De novo European eel transcriptome provides insights into the evolutionary history of duplicated genes in teleost lineages. *PloS one* **14**: e0218085.

**Sanderson MJ**. r8s: inferring absolute rates of molecular evolution and divergence times in the absence of a molecular clock. *Bioinformatics*. 2003 Jan 22;19(2):301-2.

**Schiavinato M, Marcet-Houben M, Dohm JC, Gabaldón T, Himmelbauer H**. **2020**. Parental origin of the allotetraploid tobacco Nicotiana benthamiana. *The Plant journal: for cell and molecular biology* **102**: 541–554.

**Shingate P, Ravi V, Prasad A, Tay B-H, Garg KM, Chattopadhyay B, Yap L-M, Rheindt FE, Venkatesh B**. **2020**. Chromosome-level assembly of the horseshoe crab genome provides insights into its genome evolution. *Nature communications* **11**: 2322.

**Slater GSC, Birney E**. **2005**. Automated generation of heuristics for biological sequence comparison. *BMC bioinformatics* **6**: 31.

**Smith JM, Smith NH**. **1996**. Synonymous nucleotide divergence: What is ‘saturation’? *Genetics* **142**: 1033–1036.

**Smith SA, O’Meara BC.** treePL: divergence time estimation using penalized likelihood for large phylogenies. *Bioinformatics*. 2012 Oct 15;28(20):2689-90.

**Stamatakis A.** RAxML version 8: a tool for phylogenetic analysis and post-analysis of large phylogenies. *Bioinformatics*. 2014 May 1;30(9):1312-3.

**Stanke M, Keller O, Gunduz I, Hayes A, Waack S, Morgenstern B**. **2006**. AUGUSTUS: ab initio prediction of alternative transcripts. *Nucleic acids research* **34**: W435–9.

**Suchard MA, Lemey P, Baele G, Ayres DL, Drummond AJ, Rambaut A**. **2018**. Bayesian phylogenetic and phylodynamic data integration using BEAST 1.10. *Virus evolution* **4**: vey016.

**Sun M, Naeem R, Su J-X, Cao Z-Y, Burleigh JG, Soltis PS, Soltis DE, Chen Z-D**. **2016**. Phylogeny of theRosidae: A dense taxon sampling analysis. *Journal of systematics and evolution* **54**: 363–391.

**Tang H, Wang X, Bowers JE, Ming R, Alam M, Paterson AH**. **2008**. Unraveling ancient hexaploidy through multiply-aligned angiosperm gene maps. *Genome research* **18**: 1944–1954.

**Tiffney BH**. **1986**. Fruit and Seed Dispersal and the Evolution of the Hamamelidae. *Annals of the Missouri Botanical Garden. Missouri Botanical Garden* **73**: 394–416.

**Trapnell C, Williams BA, Pertea G, Mortazavi A, Kwan G, van Baren MJ, Salzberg SL, Wold BJ, Pachter L**. **2010**. Transcript assembly and quantification by RNA-Seq reveals unannotated transcripts and isoform switching during cell differentiation. *Nature biotechnology* **28**: 511–515.

**Unver T, Wu Z, Sterck L, Turktas M, Lohaus R, Li Z, Yang M, He L, Deng T, Escalante FJ, *et al.*** **2017**. Genome of wild olive and the evolution of oil biosynthesis. *Proceedings of the National Academy of Sciences of the United States of America* **114**: E9413–E9422.

**Van Bel M, Diels T, Vancaester E, Kreft L, Botzki A, Van de Peer Y, Coppens F, Vandepoele K**. **2018**. PLAZA 4.0: an integrative resource for functional, evolutionary and comparative plant genomics. *Nucleic acids research* **46**: D1190–D1196.

**Waterhouse RM, Seppey M, Simão FA, Manni M, Ioannidis P, Klioutchnikov G, Kriventseva EV, Zdobnov EM**. **2018**. BUSCO Applications from Quality Assessments to Gene Prediction and Phylogenomics. *Molecular biology and evolution* **35**: 543–548.

**Xia E, Li F, Tong W, Yang H, Wang S, Zhao J, Liu C, Gao L, Tai Y, She G, *et al.*** **2019**. The tea plant reference genome and improved gene annotation using long-read and paired-end sequencing data. *Scientific Data* **6**: 122.

**Xiang Y, Huang C-H, Hu Y, Wen J, Li S, Yi T, Chen H, Xiang J, Ma H**. **2017**. Evolution of Rosaceae Fruit Types Based on Nuclear Phylogeny in the Context of Geological Times and Genome Duplication. *Molecular biology and evolution* **34**: 262–281.

**Xiao L, Yang G, Zhang L, Yang X, Zhao S, Ji Z, Zhou Q, Hu M, Wang Y, Chen M, *et al.*** **2015**. The resurrection genome of Boea hygrometrica: A blueprint for survival of dehydration. *Proceedings of the National Academy of Sciences of the United States of America* **112**: 5833–5837.

**Xu H, Wang P, Fu Y, Zheng Y, Tang Q, Si L, You J, Zhang Z, Zhu Y, Zhou L, *et al.*** **2010**. Length of the ORF, position of the first AUG and the Kozak motif are important factors in potential dual-coding transcripts. *Cell research* **20**: 445–457.

**Xu W, Zhang L, Cunningham AB, Li S, Zhuang H, Wang Y, Liu A**. **2020**. Blue genome: chromosome-scale genome reveals the evolutionary and molecular basis of indigo biosynthesis in Strobilanthes cusia. *The Plant journal: for cell and molecular biology* **104**: 864–879.

**Yang Z, Nielsen R, Goldman N, Pedersen AM**. **2000**. Codon-substitution models for heterogeneous selection pressure at amino acid sites. *Genetics* **155**: 431–449.

**Yang JT, Preiser AL, Li Z, Weise SE, Sharkey TD**. **2016**. Triose phosphate use limitation of photosynthesis: short-term and long-term effects. *Planta* **243**: 687–698.

**Ye Z-H, Zhong R**. **2015**. Molecular control of wood formation in trees. *Journal of experimental botany* **66**: 4119–4131.

**Zerega NJC, Clement WL, Datwyler SL, Weiblen GD**. **2005**. Biogeography and divergence times in the mulberry family (Moraceae). *Molecular phylogenetics and evolution* **37**: 402–416.

**Zhang D, Easterling KA, Pitra NJ, Coles MC, Buckler ES, Bass HW, Matthews PD**. Non‐Mendelian single‐nucleotide polymorphism inheritance and atypical meiotic configurations are prevalent in hop. *The plant genome*. 2017 Nov;10(3):plantgenome2017-04.

**Zonneveld BJM, Leitch IJ, Bennett MD**. **2005**. First nuclear DNA amounts in more than 300 angiosperms. *Annals of botany* **96**: 229–244.
